# Searching for a Correlation between Interfacial Liquid Structure and Hydrodynamic Slip: A Case Study of Water/Silica Interface

**DOI:** 10.1021/acs.jpcc.6c00760

**Published:** 2026-08-03

**Authors:** Juseok Choi, Abdul Aziz Shuvo, Adri C. T. van Duin, Bladimir Ramos-Alvarado, Seong H. Kim

**Affiliations:** † Department of Chemical Engineering and Materials Research Institute, 8082 The Pennsylvania State University, University Park, Pennsylvania 16802, United States; ‡ Department of Mechanical Engineering, The Pennsylvania State University, University Park, Pennsylvania 16802, United States

## Abstract

Hydrodynamic slip at the solid–liquid interface is governed by molecular-scale interactions; however, establishing a direct correlation between interfacial liquid structure and slip behavior is challenging due to the difficulty of isolating nanoscale interfacial structures of water at the solid surface from the bulk water structure. Here, we investigate the relationship between hydrodynamic slip and interfacial water structure at the silica–water interface as a function of NaCl concentration. Vibrational sum-frequency generation (SFG) spectroscopy was used to probe interfacial water structure by deconvoluting Stern-layer and diffuse-layer contributions in the SFG response using the maximum entropy method, and liquid-cell atomic force microscopy was used to quantify the hydrodynamic slip length. Supported by molecular dynamics simulations that resolve molecular orientation and density profiles of water near the silica surface, we identify the distinct hydrogen-bonding configuration and orientation of interfacial water molecules. In the low ionic strength regime (≤10^–5^ M NaCl), there are no significant changes in both SFG spectral features and slip length. In contrast, in the high ionic strength regime (>10^–5^ M NaCl), significant changes in the water orientation in the Stern layer can be correlated with a reduction in slip length. These results indicate that hydrodynamic slip at hydrophilic, charged interfaces is governed not simply by ion presence but by ion-driven alteration of interfacial water structure.

## Graphical abstract

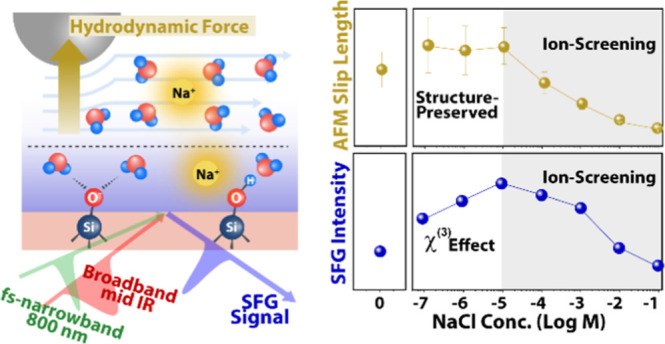

## Introduction

Although the molecular structure of water (H_2_O) is simple, its fluidic behavior is remarkably complex, especially when confined or influenced by interfaces. At the boundary between solids and liquids, the orientation distribution and dynamics of water molecules are significantly different compared to those in the bulk, which influences slip of water molecules with respect to the solid surface under hydrodynamic conditions.^1–3^ Therefore, understanding interfacial water structure is not only of scientific importance but also relevant to many technical applications, including energy conversion,^4^ catalysis,^5^ and nanofluidics.^6^

At charged solid–liquid interfaces, such as the silica–water system, the formation of an electric double layer (EDL) adds to the complexity of the interfacial water structure and its effects on hydrodynamics.^7–9^ At silica–water interfaces, the EDL forms when surface silanol groups (Si–OH) deprotonate, producing negatively charged silanolate sites (Si–O^–^) that electrostatically attract counterions from the aqueous solution.^10–12^ This interaction leads to the formation of a structured interfacial region known as the electrical double layer (EDL), which comprises the Stern layer (SL) and the diffuse layer.^13,14^ The SL consists of specifically adsorbed counterions, carrying a charge opposite to that of the surface, along with associated water molecules that are tightly bound at the interface. Beyond this region lies the diffuse layer, which contains relatively mobile ions and is characterized by an excess of countertions over ions bearing the same sign as the surface charge.^15,16^ This excess decreases gradually with increasing distance from the surface, approaching bulk concentration.^17,18^ The presence of SL and EDL not only defines the electrostatic environment at the interface but also strongly influences the orientation and hydrogen-bond network of interfacial water molecules, which directly impacts the interfacial hydrodynamic behavior.^8,9,19,20^

Hydrodynamic slip is a phenomenon in which fluids flow along solid boundaries with a nonzero surface velocity, in contrast to the no-slip boundary condition commonly used to solve the Navier–Stokes equation.^21–23^ It is sensitive to molecular-level parameters such as solid–liquid affinity, water orientation, hydrogen bonding, and density profile near the surface; collectively, they affect the availability of momentum carriers.^24,25^ At hydrophilic and charged surfaces like silica, these variables can significantly change the frictional interaction between water and the solid surface, leading to either enhanced or suppressed slippage. Despite its importance, the direct correlation between interfacial water structure and hydrodynamic slip still remains unclear due to the difficulty of distinguishing nanometer-scale interfacial water properties from those of bulk liquid water.^6,26^

The interfacial water structure can be probed with vibrational sum frequency generation (SFG) spectroscopy.^26–28^ As a second-order nonlinear optical process, SFG requires a noncentrosymmetric environment. This means that SFG is forbidden in isotropic media, such as bulk water and amorphous solids, which exhibit inversion symmetry on average and are therefore effectively centrosymmetric.^29^ However, the presence of a surface naturally creates noncentrosymmetry along the surface-normal direction, allowing SFG to selectively probe interfacial water molecules without interference from bulk water. Beyond this intrinsic surface specificity, the SFG signal is also sensitive to the net orientation of interfacial water molecules.^30–32^ For example, Nihonyanagi et al. directly measured the complex second-order susceptibility (χ^(2)^) spectra of the air/liquid interface using heterodyne-detected vibrational SFG spectroscopy (HD-VSFG).^32^ They found that the Imχ^(2)^ spectrum of pure water shows a positive band at ∼3200 cm^–1^ and a negative band at ∼3450 cm^–1^, indicating different orientations of interfacial water molecules with different hydrogen bonding interactions. In contrast, in the presence of charged surfactants, either positively charged cetyltrimethylammonium bromide (CTAB) or negatively charged sodium dodecyl sulfate (SDS), both OH bands in the Imχ^(2)^ spectrum show the same sign (either positive or negative), revealing that the interfacial water dipoles are predominantly oriented in one direction, either toward the air or the bulk, depending on the surface charge.^32^

At strongly charged surfaces, the SL and EDL contribute differently to the overall SFG signal.^16,19,31,33^ Therefore, for accurate interpretation, the SFG signals must be deconvoluted into their individual contributions. Backus and Bonn et al.^34^ and Hore and Tyrode et al.^35^ investigated the ionic strength dependence of SFG intensities at the silica/water interface, focusing on how the third-order susceptibility term (χ^(3)^), originating from water molecules in the diffuse layer, contributes to the total SFG signal. They showed that the interplay (or mismatch) between the Debye length of EDL and the coherence length of the SFG process governs the interference between the χ^(2)^ and χ^(3)^ contributions. Therefore, considering the χ^(3)^ contribution is essential for interpreting SFG spectra and interfacial water structure, especially under strongly charged conditions.

Following the analytical framework of Rehl and Gibbs,^36^ we used the Maximum Entropy Method (MEM) to analyze SFG spectra at the silica/water interface. This framework reconstructs the complex phase (real and imaginary parts) to reveal the water molecular structure. Simultaneously, it enables the separation of Stern and diffuse layer contributions by accounting for χ^(3)^ and interference effects within the electric double layer. We adopted this methodology to systematically investigate the interface and interpret SFG responses with different salt species and concentrations.

This distinction in interfacial water structure has important implications for understanding hydrodynamic slip behavior. The hydrodynamic slip length is defined as the extrapolated distance below the solid surface where the velocity profile of a liquid would vanish if a linear velocity profile in the fluid phase were extrapolated.^37^ To measure this slip length, which is typically in the nanometer scale, atomic force microscopy (AFM) has been used.^37–39^ AFM measures the drag (or drainage) forces between a spherical probe and a substrate during approach. The force–distance profile during the probe approach in AFM experiments can be modeled mathematically. Vinogradova derived the equation for the relationship between the slip length and drag force using the slippage model based on Navier’s boundary condition to describe hydrodynamic drainage between two curved surfaces.^37^

In this work, we investigated the influence of salt ions on interfacial water structure, probed with SFG, and slip length, measured with AFM, at the silica–water interface. Salt ions modify the electrostatic environment near the solid/water interface by screening surface charges and modulating the EDL thickness. Based on the imaginary second-order susceptibility, Imχ^(2)^, spectrum of the SL extracted from SFG spectra using the maximum entropy method (MEM),^7,36^ which were interpreted and supported by comparing with molecular dynamics (MD) simulation results, we found a correlation between the water molecule orientation and the hydrodynamic slip length. In the low ionic strength regime (≤10^–5^ M NaCl), the slip length is seemingly insensitive to ions, although the interfacial water structure changes. In the high ionic strength regime (>10^–5^ M NaCl), salt ions near the surface perturb the hydrogen-bond network and the orientational ordering of water molecules at the interface, which appears to correlate with the slip length decrease.

## Methods

### Sum Frequency Generation Measurement

Experimental SFG data were collected using a reflection mode with copropagation of s-polarized 800 nm (pulse width = 2 ps; 2 kHz repetition; incident angle = 45°) and p-polarized broadband IR (incident angle = 45°).^40^ A right-angle fused silica prism was used as the solid substrate, which was positioned on top of a liquid reservoir. The silica/water interface was probed from the solid side. The two copropagated incident beams entered through the rectangular lateral face of a right-angle fused silica prism (PS615, Thorlabs, USA, flatness <0.06 μm). The reflected s-polarized SFG signal was directed to the detector (DU420A-BEX2-DD with volume-phase holographic (VPH) grating, Oxford Instruments Andor). Aqueous solutions with salt concentrations ranging from 10^–7^ M to 10^–1^ M were prepared with ultrapure deionized (DI) water (18.2 MΩ·cm, Millipore Direct-Q 5 UV system, Merck, Germany), sodium chloride (S9625-500G, Sigma-Aldrich), and potassium chloride (7447-40-7, VWR). After exchanging the solution in the reservoir, a 10 min equilibration time was allowed before data collection. SFG spectra were then acquired for 15 min per broadband IR pulse. Before and after each experiment, the silica surface was cleaned using diluted sulfuric acid (258105, Sigma-Aldrich) and ammonium hydroxide (AX1303, Sigma-Aldrich) solutions to remove organic compounds and metallic ions from the surface. The cleaned surface was thoroughly rinsed with ultrapure deionized water.

### Maximum Entropy Method (MEM) to Extract the Imaginary Component of χ^(2)^ from the SFG Intensity Spectrum

To extract the complex-valued second-order nonlinear susceptibility χ^(2)^ from intensity-only SFG measurements, the MEM algorithm was used.^36^ This approach estimates the most probable phase spectrum consistent with the measured intensity spectrum by maximizing spectral entropy through variation of the phase angle. The complex susceptibility is expressed as^36^

1 $$\chi^{\left(2\right)}\left(\nu \right)=\frac{be^{i\phi_{\upsilon }}}{1+\sum_{m=1}^{M}a_{m}e^{i2\pi m\nu }}$$

where the coefficients *a*_m_ and *b* are determined by solving a Toeplitz matrix constructed from the autocorrelation function. The numerator introduces the error phase (ϕ_υ_), which varies as a function of frequency that cannot be determined by MEM alone. This error phase was estimated by comparison with the phase-sensitive SFG data reported by Myalitsin et al.^41^ A comparison of the experimental and MEM-derived complex χ^(2)^ is shown in Figure S1 in the Supporting Information.

However, despite its utility, MEM-based SFG phase retrieval requires caution, as incorrect assumptions (e.g., regarding the nonresonant background) can introduce an unintended artifacts into the extracted spectra. To avoid such artifacts, we utilized the well-established parametrization for the silica/water interface reported by Rehl and Gibbs,^36^ validating our analysis through direct comparison with their results.

### Atomic Force Microscopy Measurement

Atomic force microscopy (AFM; Digital Instrument, Multimode) was used to measure force–separation curves. AFM was equipped with a liquid cell probe holder (MTFML-V2, Bruker), which enabled probe movement and convenient solution exchange. The AFM probe was a rectangular cantilever with a spring constant of 1 N/m (Novascan), to which a 20 μm silica (SiO_2_) particle tip was glued. Solutions and flat quartz substrates (Technical Glass Products, flatness <2.0 μm) were prepared using the same chemical procedures and cleaning method as in the SFG experiments. The prepared substrates exhibited a hydrophilic surface with a contact angle near zero (Figure S2). The probe approach velocities were set to 70 μm/s for fast data collection and 0.2 μm/s for slow data collection. Based on the maximum shear rate estimated from the approaching velocity, the probe radius, the separation distant, and the slip length, the calculated Peclet number (*Pe*) is around 0.06 or smaller, confirming that our AFM measurement operates within the diffusion-dominated, quasi-equilibrium regime. This confirms that it is valid and meaningful to correlate the equilibrium SFG data with the interfacial slip under the specific nanoscale confinement and flow parameters used in this study. For each concentration, five different regions were selected, and measurements were taken at 12 points per each region (for a total of 60 points). The force–separation curves were averaged over these points for analysis with standard deviation.

### Molecular Dynamics Simulations

Equilibrium molecular dynamics (EMD) simulations were performed to characterize the molecular orientation and hydrogen-bonding distribution of interfacial water in the presence of solvated ions. All simulations were carried out using LAMMPS,^42^ and atomic trajectories were visualized using OVITO.^43^ The solid substrate consisted of an α-quartz (001) slab terminated with surface silanol groups in Figure 1a,b. A crystalline solid structure was used to enable the formation of stable and abundant silanol groups interacting with water in our size-affected model, as opposed to the amorphous quartz used in the experiments. The slab dimensions in the lateral directions were 35.4 Å × 33.4 Å, with a thickness of 15.5 Å. The bottom layer of Si and O atoms was held fixed to maintain structural stability, while all remaining atoms were allowed to have thermal vibration. A water film containing 605 molecules was placed above the quartz surface, resulting in a bulk water density of approximately 1 g cm^–3^ in Figures 1c,d. We modeled 0, 1, 3, and 5 M NaCl solutions by inserting an appropriate number of Na^+^ and Cl^–^ ions into the liquid. Note that due to the limitation in the number of water molecules in the simulation box, MD could not model the low concentration conditions; thus, we focused on the qualitative trend in concentration dependence of water orientation and hydrogen-bonding distributions. Periodic boundary conditions were applied in all directions. To eliminate the interaction between liquid atoms and the bottom solid slab atoms, a 40 Å vacuum region was included above the liquid layer. Interatomic interactions were described using the ReaxFF reactive force field, with parameters obtained by combining two established ReaxFF parameter sets. The parameter set by Hahn et al. provides a comprehensive description of silica, water, and Na^+^ species,^44^ and the parameters for Cl^‑^ ions were adopted from the work of Fedkin et al.^45^ This potential allows the simulation of chemical reactions, including bond dissociation and formation. The simulations were integrated with a 0.25 fs time step. The simulations were initialized with a 500 ps equilibration in the *NVT* ensemble at 300 K using a Nosé–Hoover thermostat.^46,47^ This was followed by a 100 ps NVE simulation to confirm the stability of the total energy, temperature, and pressure. After equilibration, a 500 ps production run was performed in the *NVE* ensemble at 300 K. Atomic trajectories were sampled every 125 fs for subsequent analysis.

**Figure 1:**
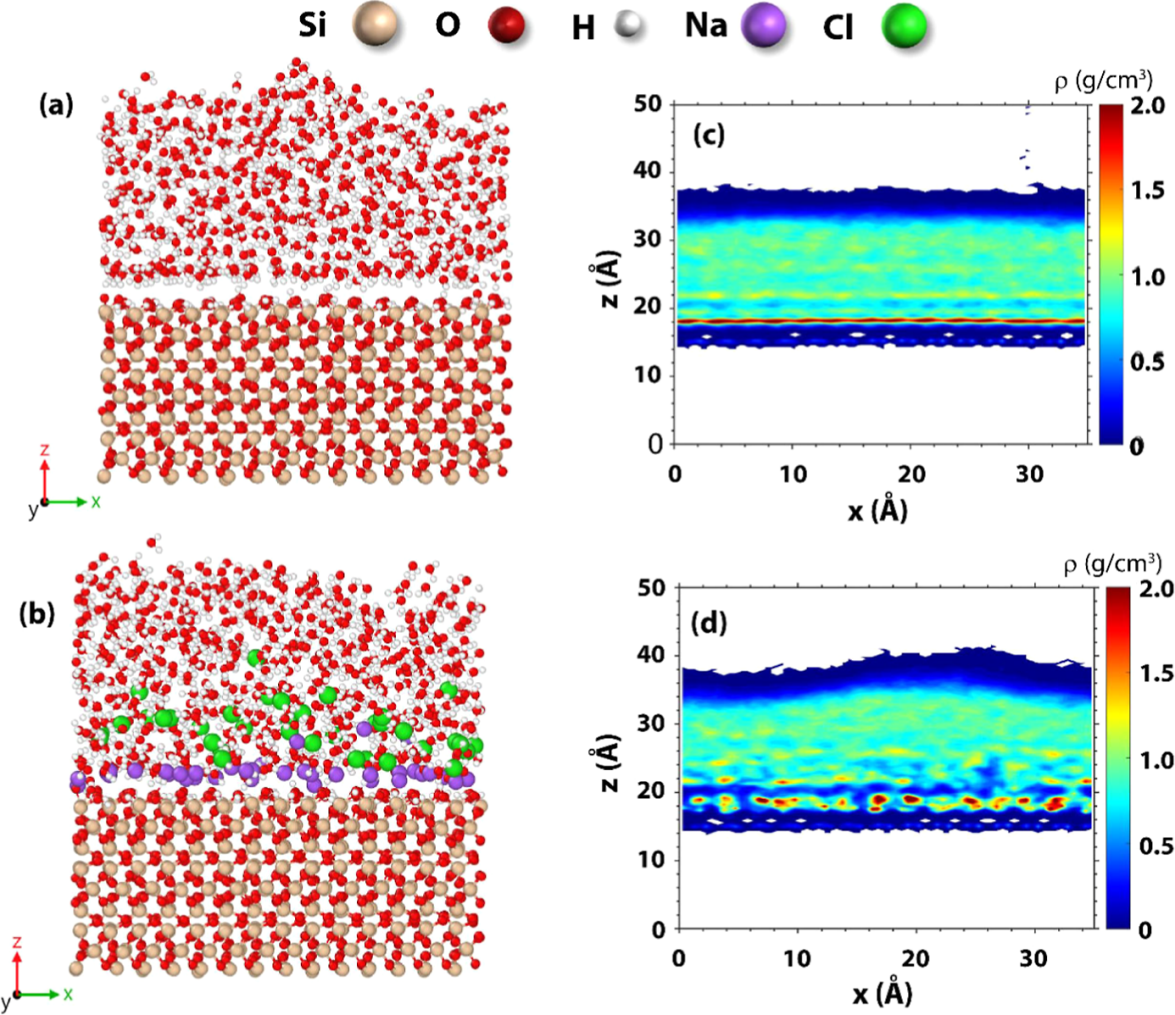
Atomistic models of the silica (α-quartz) surface in contact with (a) pure water (0 M) and (b) a 3 M NaCl solution. The right panel shows the corresponding equilibrium water-density contour plots for (c) pure water (0 M) and (d) 3 M NaCl solutions. The bulk densities of the water in (c) and (d) are approximately 1 g/cm^3^.

## Results & Discussion

### Effect of Salt Ions on Hydrodynamic Slip

Figure 2 provides an overview of the slip length measurements using liquid-cell AFM. As shown in Figure 2a, the experimental setup consists of an enclosed fluid cell, where a 20 μm SiO_2_ particle-modified AFM probe tip is submerged in liquid and contacts a quartz substrate. When the AFM probe approaches the surface in the liquid environment (Figure 2b), it experiences electrostatic repulsion and van der Waals attraction. These interfacial force components can be described with the Derjaguin–Landau–Verwey–Overbeek (DLVO) theory, as schematically shown in Figure 2c.^48,49^ The DLVO framework combines EDL repulsion from surface potentials and van der Waals attraction to predict the net interaction potential as a function of tip-surface distance. These interactions will govern the force during the slow approach. However, at high approaching velocities, an additional hydrodynamic drainage force develops from the viscous resistance to the lateral flow of the liquid being squeezed out. The hydrodynamic force, *F*_h_, acting between a spherical tip and a flat surface in a viscous fluid is given by^50^

2 $$F_{h}=\frac{6\pi \eta r^{2}v_{a}}{h}f^{*}$$

Here, η is the fluid viscosity, *r* is the sphere radius, *v*_a_ is the approach velocity, and *h* is the separation between the bottom of the sphere and the flat surface. In this study, the salt concentrations of interest ranged from 10^–7^ M to 10^–1^ M. Within this range, we can assume that the rheological properties of the solution are the same as those of pure water.^51^ Based on this assumption, we set η to 0.89 cP, the same as pure water, for slip length calculations. While the effective viscosity (η_eff_) near the interface may be different from the bulk viscosity (η_bulk_) due to surface-induced effects,^24,52^ our analysis focuses on the relative trends across concentrations rather than absolute values. Sensitivity analyses using viscosity ratios (η_eff_/η_bulk_) ranging from 1 to 2 confirm that the overall trend remains unchanged in Figure S3. The correction factor f*, derived by Vinogradova, is a function of both *h* and the slip length *L*_s_ ^50^

3 $$f^{*}=\frac{h}{3L_{s}}\left[\left(1+\frac{h}{6L_{s}}\right)\ln\left(1+\frac{6L_{s}}{h}\right)-1\right]$$

**Figure 2:**
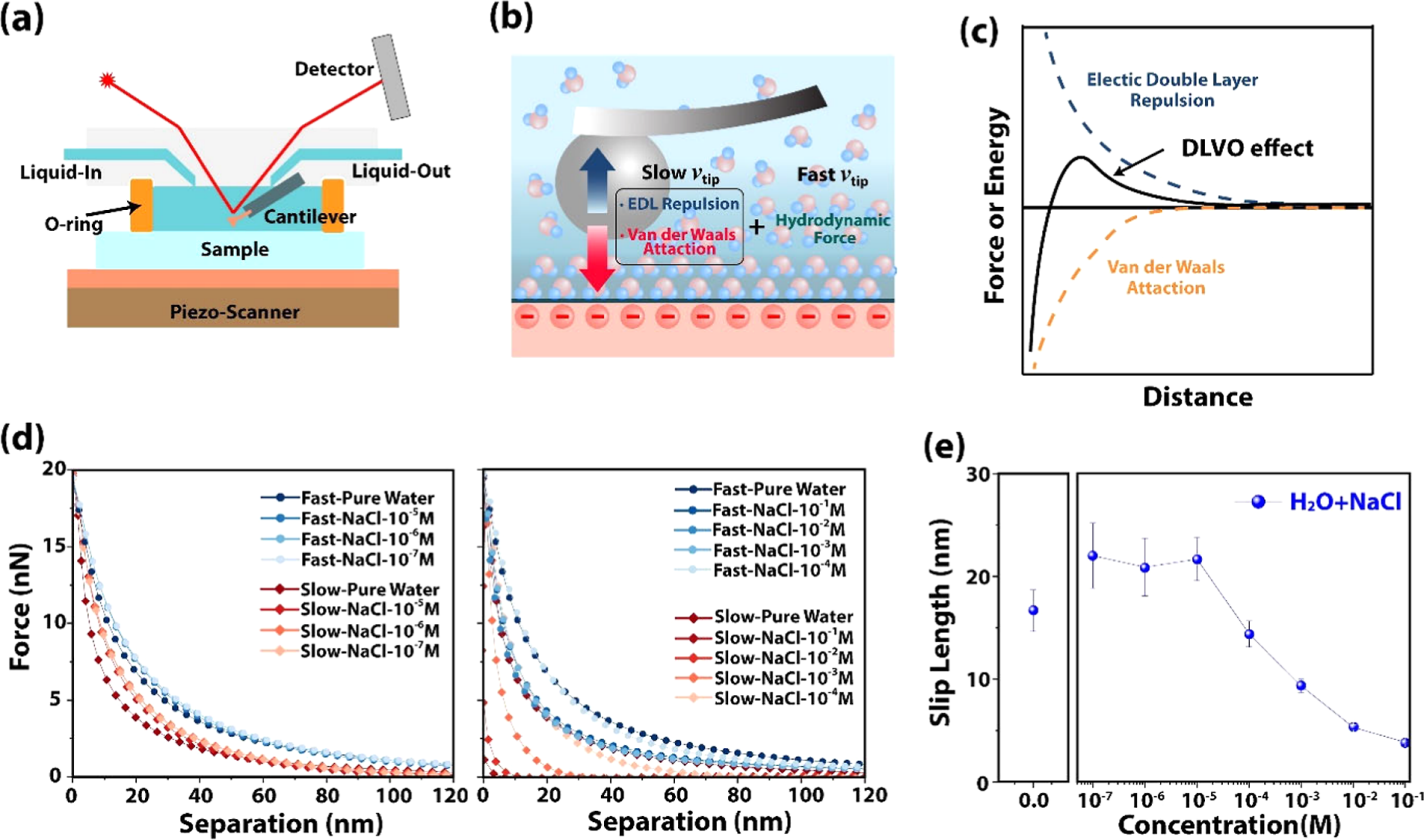
(a) Schematic of the liquid-cell AFM setup used for slip length measurements under aqueous conditions. (b) Visualization of interfacial forces acting on the AFM tip, including the electric double layer (EDL) repulsion, van der Waals attraction, and hydrodynamic drainage force. (c) Classical DLVO theory depicting the net interaction potential as a combination of EDL repulsion and van der Waals attraction. (d) Experimentally measured force–separation profiles during slow and fast AFM approach at varying NaCl concentrations (left-side panel: low-concentration, and right-side panel: high-concentration). (e) Extracted slip length as a function of NaCl concentration.

By fitting the measured force–distance data with this theoretical model, the slip length *L*_s_ can be quantitatively extracted.

Figure 2d presents the measured force–separation curves obtained from liquid-cell AFM experiments performed in water and NaCl solutions at various concentrations. In slow tip-approaching conditions (red markers), the viscous drainage force is negligible; thus, the measured force is dominated by conservative interactions (e.g., DLVO). In comparison, in fast tip-approaching conditions (blue markers), hydrodynamic drainage forces become dominant. To isolate the hydrodynamic contribution, the force data collected under the fast approach conditions were corrected by subtracting the corresponding slow-approach measurements. Lastly, the slip length was calculated by fitting the corrected force–separation curves using eq 3.

Figure 2e shows a clear decrease in slip length with increasing NaCl concentration. In pure water and low ionic strength conditions (≤10^–5^ M), slip lengths are close to ∼20 nm. However, as the salt concentration increases above 10^–5^ M, the slip length significantly decreases, reaching a value below 5 nm at 0.1 M NaCl. This trend infers that ion-induced friction or structural reorganization at the interface increases at high concentrations (>10^–5^ M), leading to more no-slip-like boundary behavior. Similar experiments performed with KCl show a qualitatively identical trend (Figure S4). This implies that the observed slip behavior is not an isolated phenomenon specific to Na^+^, but rather potentially applicable to other monovalent electrolytes.

### Correlation between the SFG Intensity and the Slip Length

Figure 3a shows the ssp-polarized SFG spectra (with s-polarized SFG, 800 nm visible, and p-polarized IR beams, respectively) of the silica/water interface as a function of NaCl concentration, ranging from pure water to 0.1 M at pH 7. The spectra were collected in the OH stretching vibration region, where interfacial water molecules show vibrational modes sensitive to hydrogen bonding strength and molecular orientation.^12,26,53,54^ Two characteristic peaks near 3200 cm^–1^ and 3400 cm^–1^ are consistently observed at all concentrations. These features have been frequently interpreted in earlier studies as the signatures of two distinct water structures: the 3200 cm^–1^ band is typically assigned to strongly hydrogen-bonded (ice-like) water structures, whereas the 3400 cm^–1^ band is attributed to weakly hydrogen-bonded (liquid-like) water structures.^26^

**Figure 3:**
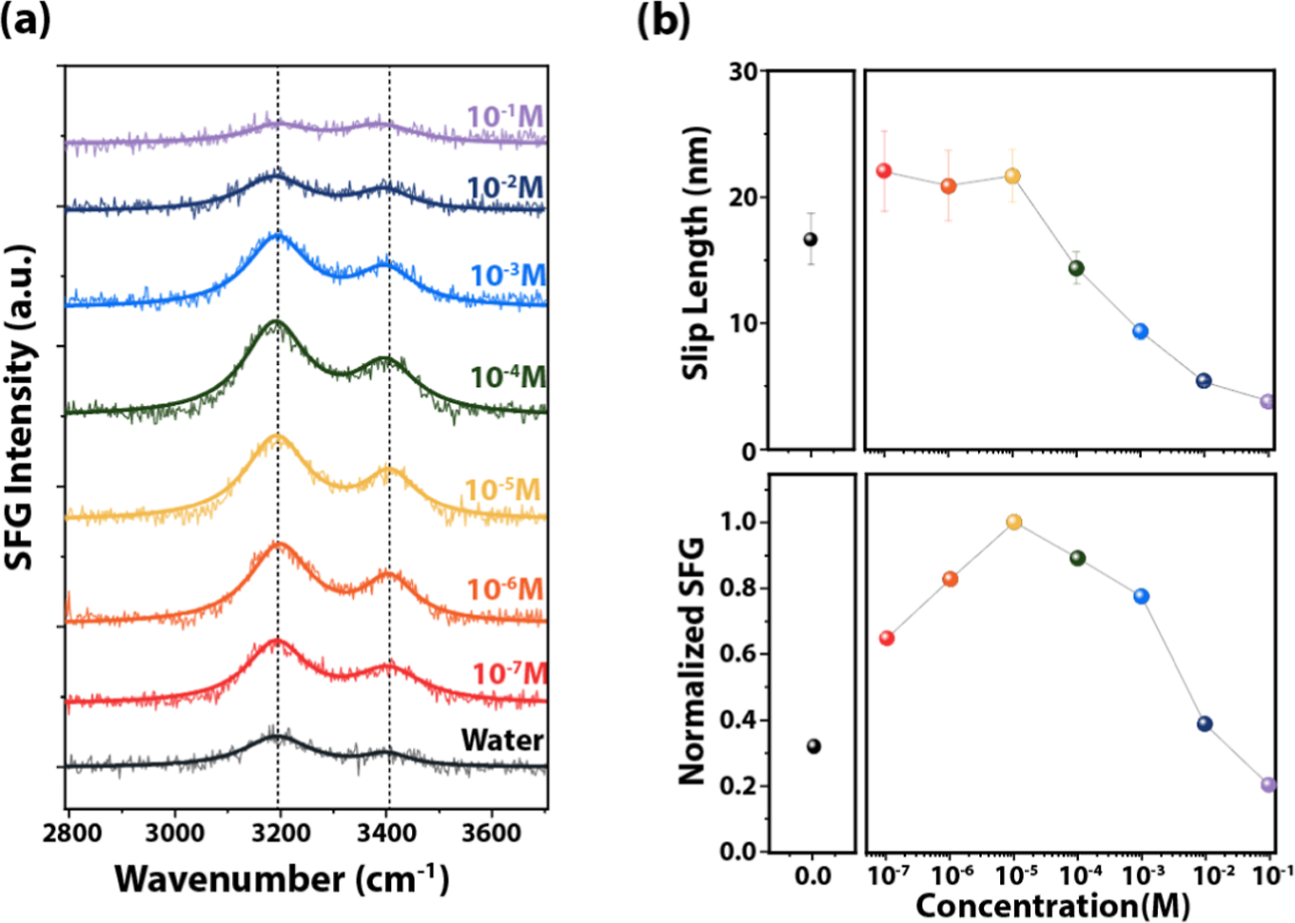
(a) SFG spectra of the silica/water interface collected in ssp polarization (SFG, Vis, IR) at pH 7, with NaCl concentrations ranging from pure water to 0.1 M. (b) Comparison of the slip length, replotted from Figure 2e, and the total SFG intensity as a function of NaCl concentration.

As shown in Figure 3b, the overall SFG intensity initially increases with salt concentration, reaching a maximum at 10^–5^ M NaCl, but gradually decreases as the salt concentration increases further. The trend in the high concentration regime appears to correlate with the measured slip length. This behavior suggests a potential link between interfacial water structure probed with SFG and hydrodynamic slip behavior probed with AFM. However, simply comparing the total SFG intensity to slip length does not reveal the relationship between interfacial water structure (especially, hydrogen-bonding and molecular orientation) and the interfacial slip. To explore this connection more meaningfully and accurately, the SFG spectral response needs to be effectively interpreted by incorporating structurally sensitive metrics, such as the imaginary part of the second-order susceptibility, Imχ^(2)^, in conjunction with MD simulations. The MD results provide complementary molecular-scale insight into interfacial water structure and ion behavior, helping to interpret spectral features that are often challenging to isolate experimentally.

### Molecular Water Structure at Silica/Water Interface

The nonmonotonic trend of the SFG spectral features as a function of NaCl concentration (Figure 3a) can be explained by the nonlinear contributions from the EDL layer.^35,36,55^ To obtain the net second-order susceptibility, all SFG spectra were reconstructed by dividing the Fresnel coefficients corresponding to the laser configuration in this study.^36^ At charged interfaces like silica at pH 7, the total second-order susceptibility contributing to the SFG signal is made up of two main sources. One is the surface-bound response χ^(2)^ from the SL (χ_Stern_^(2)^), and the other is the diffuse layer contribution (χ_Diffuse_^(2)^), which originates from water molecules influenced by the electrostatic field decaying from the surface into the bulk. The χ_Diffuse_^(2)^ can be described as

4 $$\chi_{Diffuse}^{\left(2\right)}=\chi^{\left(3\right)}\times g_{3}$$

where χ^(3)^ is the third-order susceptibility of water in the diffuse layer, and *g*_3_ is an interference term that accounts for the phase mismatch between the generated SFG signal from the preferentially aligned water molecules in the diffuse layer within the coherence length. The *g*_3_ term depends on the Debye length, coherence length, and interfacial potential as indicated in eq 5 ^36^

5 $$g_{3}=\Phi_{0}-\frac{4ik_{b}T\Delta k}{e}\sum_{n=1}^{\infty }\frac{1}{\left(2n-1\right)\left(i\Delta k-\kappa \left(2n-1\right)\right)}{\left(\frac{e\Phi_{0}}{4k_{b}T}\right)}^{2n-1}$$

where Φ_0_, *k*_b_, *T*, *e*, κ, and Δ*k* are the surface potential, Boltzmann constant, temperature, elementary charge, inverse of Debye length, and phase mismatch, respectively. Although the details of the interfacial potential can vary depending on the electrostatic model used to describe the EDL,^36^ for simplification, we used the empirical equation from a previous study describing surface potential as a function of NaCl concentration at the silica/water interface at pH 7 using the zeta potential and the Grahame equation.^56,57^ Thus, *g*_3_ can be calculated with a given set of input parameters for laser configurations and salt concentrations. The *g*_3_ calculated as a function of NaCl concentration for our experimental geometry is shown in Figure S5.

At low ionic strength, the Debye length is large, and the diffuse layer extends far into the bulk. Under these conditions, the generated SFG signal from water contributes to a partial phase-match.^35^ However, when the EDL thickness decreases and becomes comparable to the coherence length, the SFG signal comes from the fully phase-matching region. With further salt additions, the Debye length gets even smaller than the SFG coherence length. Thus, the reduction of detected volume results in a decrease in *g*_3_ as well as the SFG response.^7,35,36^ These considerations conceptually explain why the overall SFG intensity is substantially influenced by the diffuse layer contribution, which is convoluted with the spectroscopic response from the SL.

To quantify the contribution of the diffuse layer and the Stern layer to the total nonlinear susceptibility, the following equation can be used^36^

6 $$\chi_{total}^{\left(2\right)}=\chi_{Stern}^{\left(2\right)}+\chi_{Diffuse}^{\left(2\right)}=\chi_{Stern}^{\left(2\right)}+\chi^{\left(3\right)}\times g_{3}$$

Here, χ_total_^(2)^ can be obtained from the experimental data (Figure 3a) by taking the square root of the SFG signal intensity normalized with the input 800 nm, IR beam intensities, and Fresnel coefficients,^40^ and *g*_3_ can be calculated using eq 5 (Figure S5). So, once the third-order susceptibility term χ^(3)^ is determined, the contribution from the interfacial water in the SL, χ_Stern_^(2)^, can be calculated. For that purpose, it was assumed that χ_Stern_^(2)^ is constant in the high ionic strength regime.^36^ The χ_total_^(2)^ difference between 10^–3^ M and 10^–1^ M was divided with the difference in their corresponding g_3_ values to obtain the χ^(3)^ spectrum. The calculated χ^(3)^ shown in Figure S6 is consistent with the reported data.^36,55^

Figure 4a,b show the imaginary part of the second-order susceptibility for the diffuse layer (Imχ_Diffuse_^(2)^) and Stern layer (Imχ_Stern_^(2)^), respectively, at the silica/water interface in pure water, as extracted using the MEM algorithm.^36,58^ The real component spectra are shown in Figure S7. The imaginary part of the χ^(2)^ spectrum is especially useful because it reflects the resonant signals as well as molecular orientation information, while the real part is from dispersive interferences, which cannot be directly linked to structural information. In our experiment, the surface was probed from the solid side, and the detector was on the solid side. In this geometry, Imχ^(2)^ >0 means the electric dipole moment of water is facing the surface, in other words, the hydrogen atoms of water molecules are facing the substrate, while Imχ^(2)^ <0 indicates the water dipole pointing away from the surface, which means the hydrogen atoms are pointing toward the liquid phase.

**Figure 4:**
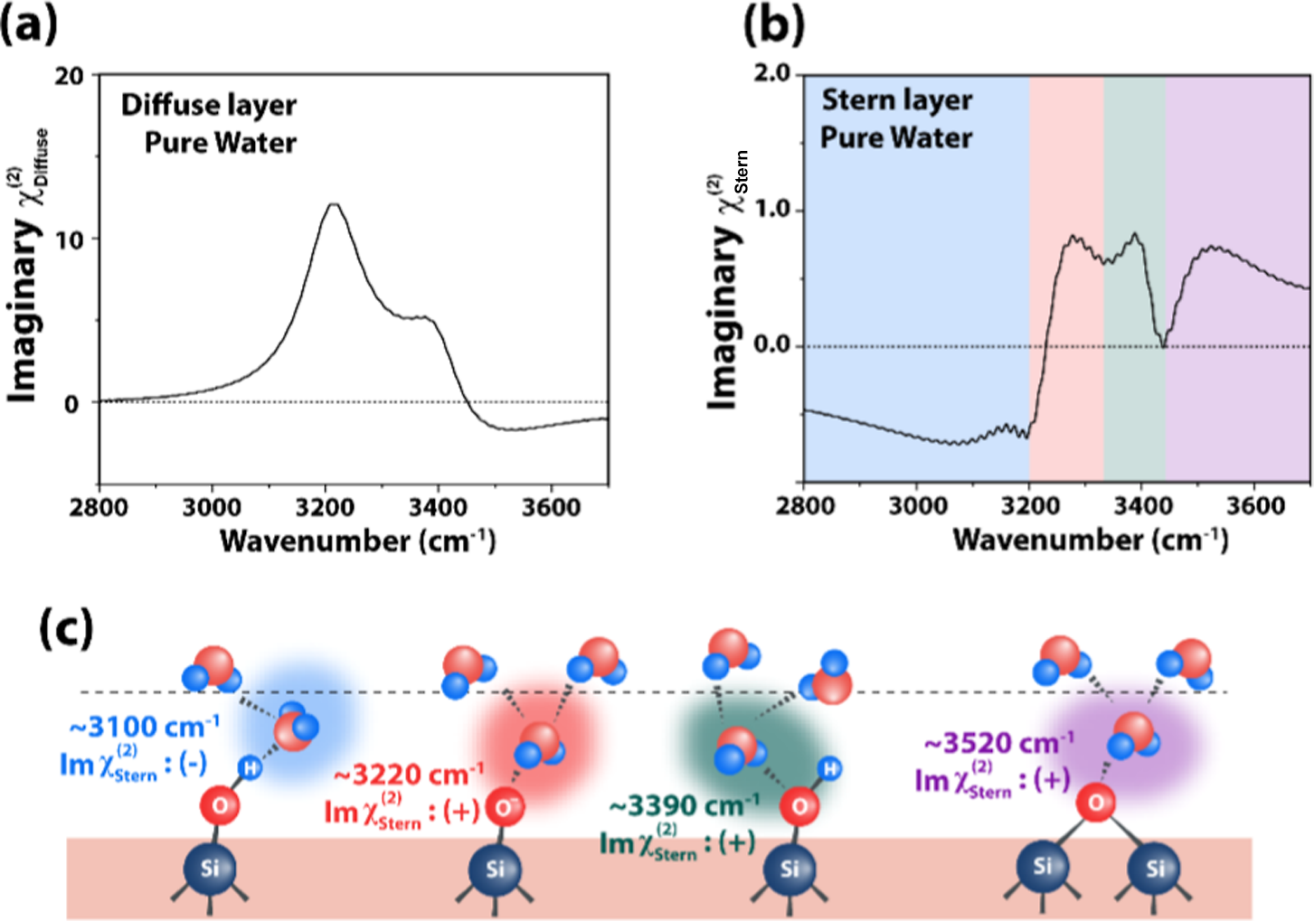
MEM-derived spectra (a) Imχ_Diffuse_^(2)^ and (b) Imχ_Stern_^(2)^ for pure water in vicinity of silica. In (b), the main vibrational features (∼3100, 3220, 3390, and 3520 cm^–1^) used for spectral interpretation are highlighted with the colored region (blue, orange, green, and purple, respectively). (c) Proposed peak assignments in the Stern layer Imχ^(2)^ spectrum based on interfacial hydrogen bonding interactions between water molecules and silica surface functional groups. The dotted line can be considered the boundary between the Stern layer and the diffuse layer, and the orientations of water molecules above the dotted line are schematically drawn based on the sign of the diffuse layer Imχ^(2)^ spectrum shown in (a).

The spectrum in Figure 4a shows two strong positive peaks near 3200 and 3400 cm^–1^, attributed to water molecules in the diffuse layer that are aligned by the electrostatic field from the negatively charged silica surface. Because partially positively charged hydrogen atoms of water are attracted toward the surface, the net dipole orientation of water molecules in the diffuse layer points to the silica surface, giving positive Imχ_Diffuse_^(2)^. Note that this net dipole simply means inequality in the population of water molecules pointing in two opposite directions. A small negative peak near 3500 cm^–1^ suggests the presence of a minor population of water molecules with opposite orientation, which is possibly from localized structural heterogeneity within the diffuse layer.^19,36^

Figure 4b shows the Imχ_Stern_^(2)^ spectrum of water molecules in the Stern layer with four dominant peaks centered around ∼3100, 3220, 3390, and 3520 cm^–1^. First, it is important to consider that the Stern layer signal cannot originate from internal hydrogen bonding between adjacent surface silanol groups. Due to the structural constraints of the Si–O–Si bond length and angle in silica, vicinal silanol–silanol interactions are spatially too far to form stable hydrogen bonds to each other. Therefore, all observed features are most reasonably attributed to interfacial water molecules that are hydrogen-bonded to surface functional groups—residual undissociated silanol (Si–OH), siloxane (Si–O–Si; a.k.a. bridging oxygen), and silanolate (Si–O^–^).

The broad negative peak near 3100 cm^–1^ (highlighted with a blue background) is attributed to water molecules that accept hydrogen bonds from surface silanol groups. In this configuration, the electric dipoles of both the water molecule (H-acceptor) and the silanol group (H-donor) point away from the silica surface, resulting in a net dipole moment oriented to the bulk water. This geometry gives a negative Imχ^(2)^ signal, drawn in the first schematic of molecular geometry (blue) in Figure 4c.

In contrast, the positive peak near 3220 cm^–1^ is assigned to water molecules that donate hydrogen bonds to silanolate (Si–O^–^) groups on the surface. In this case, the hydrogen atom of the water molecule faces the surface, producing a positive Imχ^(2)^ value (second molecular geometry (red) in Figure 4c). The positive band centered around 3390 cm^–1^ is likely due to water molecules donating hydrogen bonds to nonionized silanol oxygens, resulting in weaker hydrogen-bonding interactions than water molecules attached to the silanolate groups; they will also have a positive Imχ^(2)^ signal (third one (green) in Figure 4c). Lastly, the broad peak near 3520 cm^–1^ is consistent with very weak or nonconventional hydrogen bonding, possibly involving interactions between water molecules and siloxane bridges (Si–O–Si) (fourth one (purple) in Figure 4c).

To verify the SFG analysis and directly visualize interfacial water orientations, we performed MD simulations of the α-quartz/water interface. Note that the simulated quartz surface consists only of silanol groups (Si–OH) and bridging oxygens (Si–O–Si), as the ReaxFF force field does not allow the formation or retention of silanolate groups (Si–O^–^) at the surface in contact with water molecules. As illustrated in Figure 5, the MD simulations capture two distinct interfacial hydrogen-bonding configurations: water molecules that accept a hydrogen bond from silanol groups (Figure 5a), and water molecules that donate a hydrogen bond to silanol groups (Figure 5b). At the α-quartz (001) surface, all bridging oxygens point inward (which can be seen more clearly in Figure S9). Consequently, our ReaxFF-MD simulations do not account for water molecules hydrogen-bonded to bridging oxygen atoms, which are likely present on amorphous silica surfaces.

**Figure 5:**
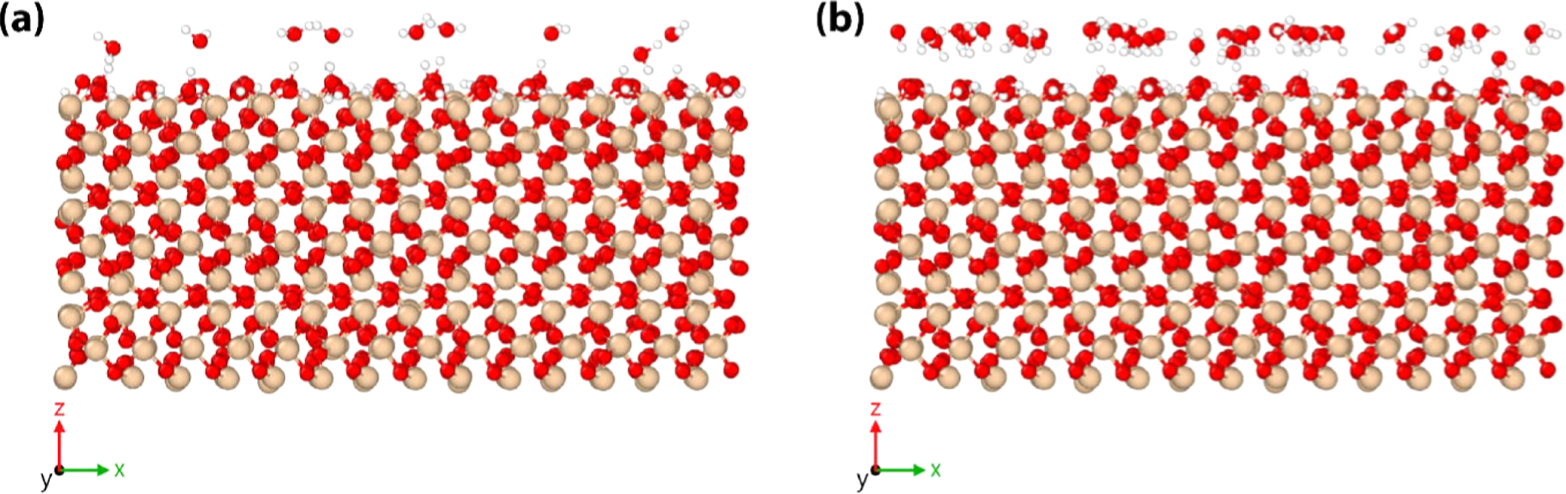
Interfacial water orientation at the α-quartz (001)/water interface. (a) Orientation of water molecules (H-acceptor) that accept hydrogen bonds from surface silanol groups (H-donor), associated with the SFG feature near 3100 cm^–1^, and (b) orientation of water molecules (H-donor) that donate hydrogen bonds to surface silanol oxygens (H-acceptor), corresponding to the SFG feature near 3390 cm^–1^. The views from the *x*-axis are shown in the Supporting Information (Figure S8).

Because the OH stretching peak position is related to the hydrogen-bond strength, we can identify which experimentally observed peaks correspond to the two orientations found in MD simulations within specific coordination geometries. We computed the hydrogen-bond lengths associated with the two OH stretching features and compared them with experimental data and empirical correlations.^59–61^ Figure 6 shows that the water molecules accepting a hydrogen bond from silanol groups (Figure 5a) correspond to the ∼3100 cm^–1^ species observed in the experimental spectra in Figure 4b, while water molecules donating a hydrogen bond to silanol groups (Figure 5b) correspond to the ∼3390 cm^–1^ species observed in Figure 4b. This corroborates the assignment of the 3220 cm^–1^ peak to the water molecules donating a hydrogen bond to silanolate groups since they will have stronger hydrogen bonding than the ones interacting with silanol groups.

**Figure 6:**
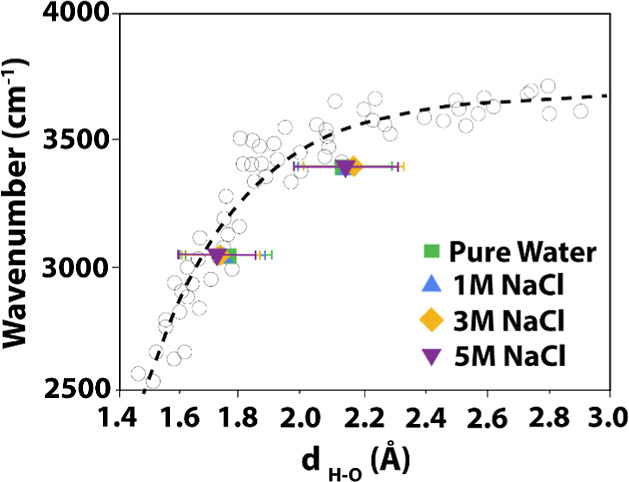
Relationship between SFG peak positions and hydrogen-bond length for different NaCl concentrations. Experimental data (dots) and the fitting result (dotted line) were extracted from the published figure in^59^ and replotted together with the present results. Adapted from^59^. Available under a CC BY 4.0 license. Copyright 2020 Dien Ngo et al.

The orientations of these interfacial water molecules were further quantified by computing the angles of the C_2v_ symmetry axis of water (θ_Ow_) and the angle between the two hydrogen atoms (θ_HH_) with respect to the surface normal direction, as illustrated in Figure 7a. The joint probability distributions of (θ_Ow_, θ_HH_), shown in Figure 7b,c, reveal clear differences in orientation between the two hydrogen-bonding arrangements. For the ∼3100 cm^–1^ case, where water accepts a hydrogen bond from the silanol group, the dominant configurations have 90° <θ_Ow_ <135°, indicating that the hydrogen atoms of water tend to point away from the surface, see Figure 7b. In contrast, for the ∼3390 cm^–1^ case, where water donates a hydrogen bond to the silanol oxygen, the preferred orientations exhibit 30° <θ_Ow_ <90°, meaning the hydrogen atoms of water are directed toward the surface, see Figure 7c. These orientation trends from MD corroborate the results from the MEM analysis of the experimental SFG spectra, confirming the molecular-level interpretation of the observed spectral features (Figure 4c). MD-derived surface charge and electrostatic potential maps (Figures S10 and S11) support the concentration-dependent changes in interfacial water structure by showing increasingly heterogeneous and cation-screened interfacial environments.

**Figure 7:**
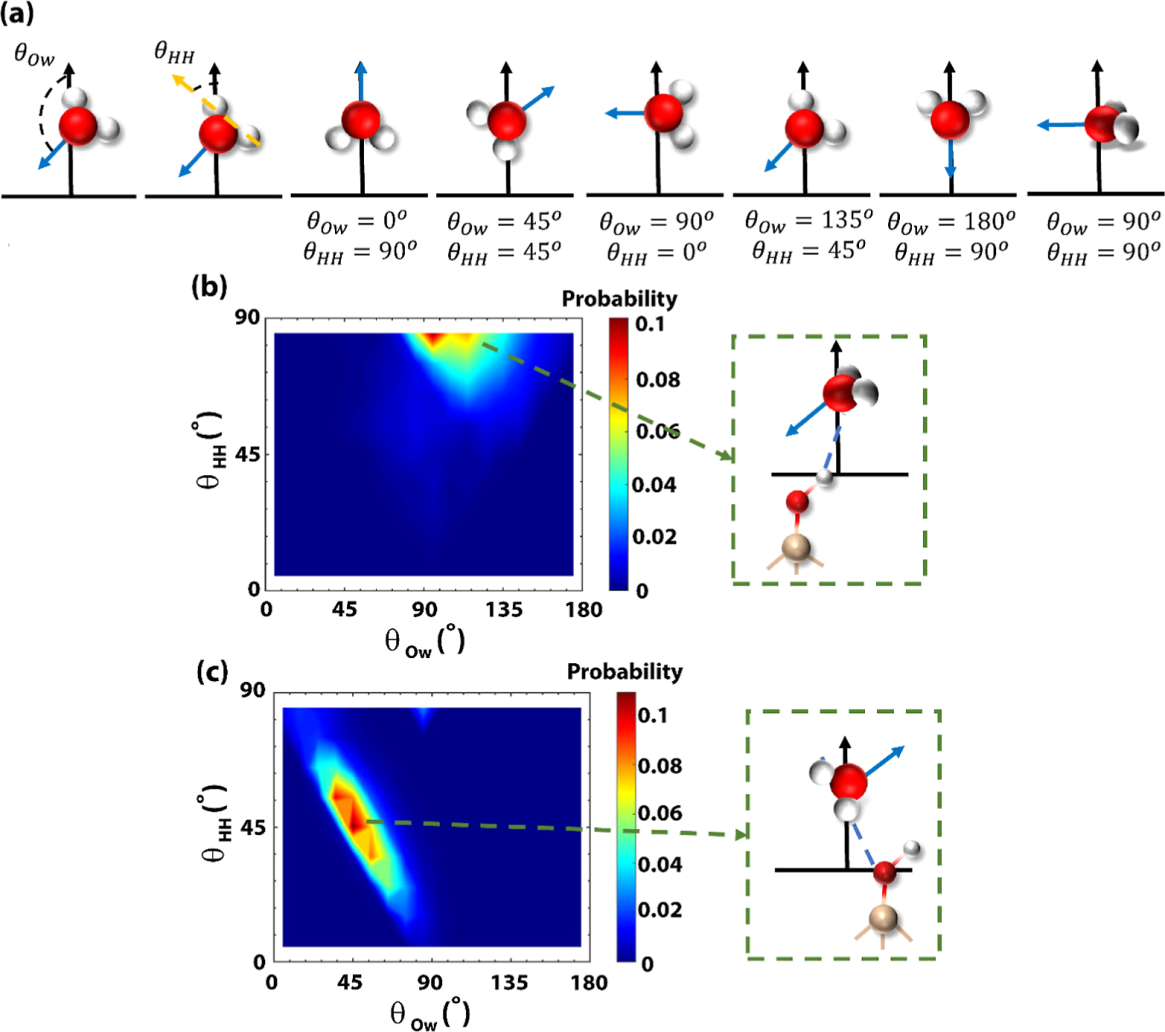
(a) Schematic representation of the orientational angles θ_Ow_ and θ_HH_, (b) joint probability distribution of θ_Ow_ and θ_HH_ for the case where the water molecule acts as the H-acceptor and the silanol group acts as the H-donor, and (c) joint probability distribution of θ_Ow_ and θ_HH_ for the case where the water molecule acts as the H-donor and the silanol group acts as the H-acceptor. Note that the blue arrow coming out of the oxygen atom is the *C*_2*v*_ symmetry axis; the electric dipole of water molecule is opposite to this arrow.

Although the ∼3220 cm^–1^ species (Figure 4c) could not be simulated with ReaxFF force fields, it is expected that the corresponding water molecule has the same orientation distribution as the ∼3390 cm^–1^ species because it donates a hydrogen bond to the silanolate group. Indeed, the MEM analysis of the experimental spectra shows that its phase is the same as the ∼3390 cm^–1^ peak (Figure 4b). Similarly, the ∼3520 cm^–1^ species hydrogen-bonded to the bridging oxygen (Figure 4c) is expected to have the same phase as the ∼3390 cm^–1^ species (Figure 4b).

### Effect of Salt Ions on the Interfacial Water Structure

Having established the spectral features of the Stern and diffuse layers at the silica/water interface for pure water (Figure 4) supported by MD simulations, we have investigated how these features change upon the addition of salt, especially the effect of the NaCl concentration in the bulk phase on the interfacial hydrogen bond interaction in the Stern layer (SL). Figure 8 displays the Imχ_Stern_^(2)^ spectra obtained for NaCl concentrations varying from 10^–7^ M to 10^–1^ M. Overall, the data could be grouped into two concentration regimes: low ionic strength (≤10^–5^ M) and high ionic strength (>10^–5^ M), to highlight the distinct spectral behaviors observed.

**Figure 8:**
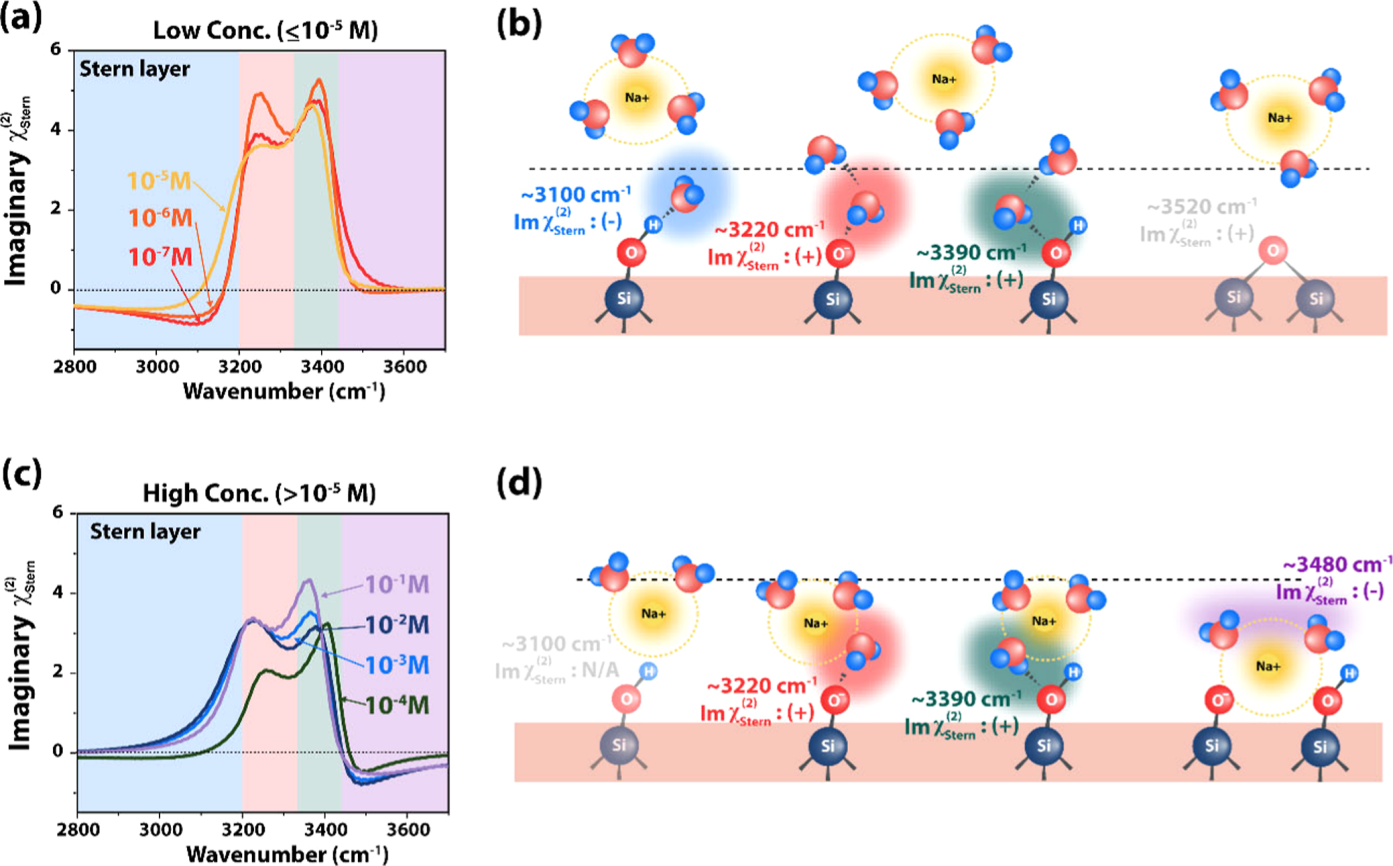
Imaginary component of χ_Stern_^(2)^ and schematic illustration of interfacial water structures (a,b) at low NaCl concentrations (≤10^–5^ M) and (c,d) at high concentrations (>10^–5^ M).

In the low concentration regime (Figure 8a), the Imχ_Stern_^(2)^ spectra show three dominant peaks centered near 3100, 3220, and 3390 cm^–1^, indicating relatively insignificant perturbation to the Stern layer water structure compared to pure water. The exception is the 3520 cm^–1^ component, and its disappearance could be due to the weak interaction with the surface (bridging oxygens, Figure 8b). These results imply that the population of positively charged Na^+^ ions in the Stern layer has not yet increased significantly.

In contrast, at higher NaCl concentrations (Figure 8c), the spectral features change significantly. The 3100 cm^–1^ peak observed at low concentrations disappears. The disappearance of the ∼3100 cm^–1^ peak at high concentrations can be attributed to the presence of Na^+^ ions in the Stern layer, replacing water molecules that accept hydrogen bonds from silanol groups. The 3220 and 3390 cm^–1^ peaks remain strong across all higher concentrations, though their intensities are reduced. A new peak also appears near 3480 cm^–1^. While this new spectral region is mostly governed by the tail of the positive 3390 cm^–1^ band at low concentrations, it became negative at higher concentrations, indicating a flip in the net orientation of the corresponding water molecules. Again, this could be attributed to the ingress of Na^+^ ions into the Stern layer; then, water molecules interacting with such positive charges will produce the negative signal in the Imχ_Stern_^(2)^ spectrum since their OH dipoles are pointing away from the surface (Figure 8d). It is also possible that the local accumulation of positive ions near the surface may shift the acid–base equilibrium of the silica surface, promoting deprotonation of silanol groups into silanolate (Si–O^–^). If so, this will further support the disappearance of the ∼3100 cm^–1^ in the high ionic strength regime.

In the high concentration regime, the vibrational modes near 3220 and 3390 cm^–1^ remain prominent. This implies that Na^+^ ions cannot fully displace water molecules in the hydrogen-bonded to the silanolate groups and the nonionized oxygen of silanol groups. Those surface-bound water molecules may be involved in the lower half of the solvation shell for the Na^+^ ions in the Stern layer. Due to the strong electric field gradient in the Stern layer, the water molecules in the upper half of the Na^+^ solvation shell may have slightly different interactions from those in the lower half. If so, these lower positioned species could be detected at a different peak position in the SFG spectrum. In a similar view, we speculate that water molecules in the upper half of the Na^+^ solvation shell might correspond to the negative ∼3480 cm^–1^ peak, although the physical origin of water species corresponding to this peak cannot be fully explained based on the current data. Such water molecules are expected to orient with the oxygen atoms pointing toward the Na^+^ ions in the Stern layer, resulting in the hydrogen atoms pointing in the bulk direction and a negative sign in Imχ^(2)^ (Figure 8d).

To further investigate how the ions influence interfacial water orientation, MD simulations were performed for 1, 3, and 5 M NaCl solutions. These concentrations are obviously higher than those used experimentally; but due to the limited number of water molecules in the model (Figure 1), the low concentration cases could not be simulated. Nonetheless, we believe MD simulations can still provide qualitative insights into the local structure of water molecules perturbed by Na^+^ ions.

Figure 9 illustrates the joint probability distributions for two interfacial hydrogen-bonding arrangements associated with the SFG features near 3100 cm^–1^ and 3390 cm^–1^. As shown in Figure 9a,b, at 1 M NaCl, the orientational preferences of interfacial water molecules remain unchanged from the pure-water case for both peaks (Figure 7b,c). However, at higher concentrations (3 and 5 M), deviations from pure-water and low-concentration regimes emerge. For the ∼3100 cm^–1^ corresponding to water molecules acting as H-acceptors, the probability of configurations with θ_Ow_ >135° increases slightly (Figure 9c,e), indicating a stronger tendency for the C_2v_ molecular axis of water to tilt toward the surface. In contrast, for the ∼3390 cm^–1^ corresponding to water molecules acting as H-donors (Figure 9d,f), the C_2v_ molecular axis distribution tilts toward the surface parallel direction (θ_Ow_ → 90°) and the molecular plane is aligned with the surface-normal direction (θ_Ow_ → 0°); see schematics in Figure 7a.

**Figure 9:**
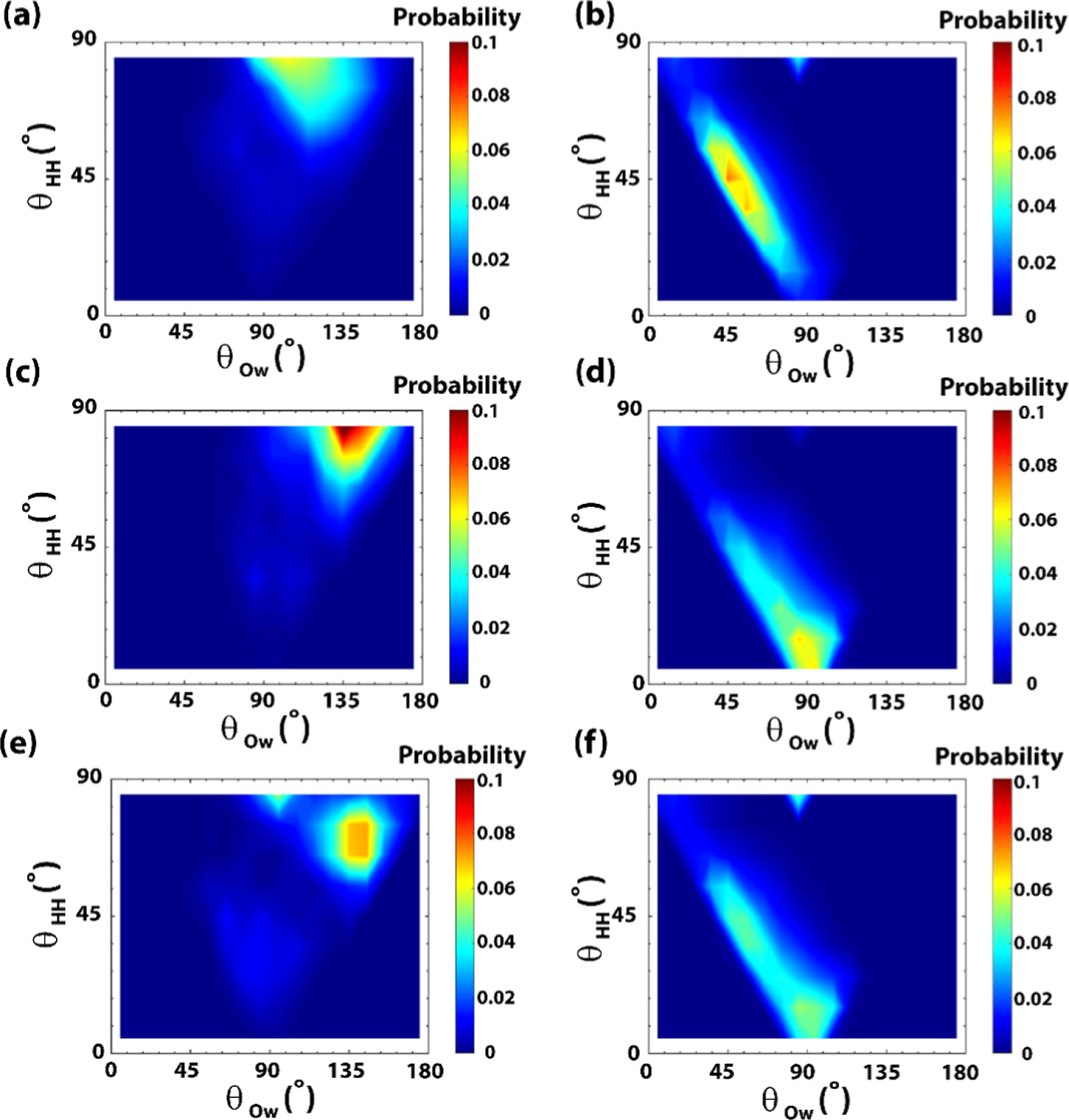
Effect of salt concentration on interfacial water orientation. Joint probability distributions of θ_Ow_ and θ_HH_ for NaCl solutions at (a,b) 1 M, (c,d) 3 M, and (e,f) 5 M NaCl solutions. Panels in the left column correspond to the interfacial hydrogen-bond arrangement in which water acts as an H-acceptor (SFG feature near 3100 cm^–1^), while panels in the right column correspond to the arrangement in which water acts as an H-donor to the silanol group (feature near 3390 cm^–1^).

MD simulations were used to evaluate the population of interfacial water molecules associated with each OH stretching feature (Figure 10). For 3100 cm^–1^, the hydrogen-bond population is similar for pure water and 1 M NaCl but slightly decreases at 3 and 5 M. However, because the absolute population of these configurations is so low, the decrease observed at higher salt concentrations could be within statistical noise of the small sampling size. For the 3390 cm^–1^ feature, a clearer trend is observed, with the population decreasing as the salt concentration increases. This trend is qualitatively consistent with the decrease of the positive peak intensities in the Imχ_Stern_^(2)^ spectra when transitioning from the low-concentration regime (Figure 8a) to the high-concentration regime (Figure 8c).

**Figure 10:**
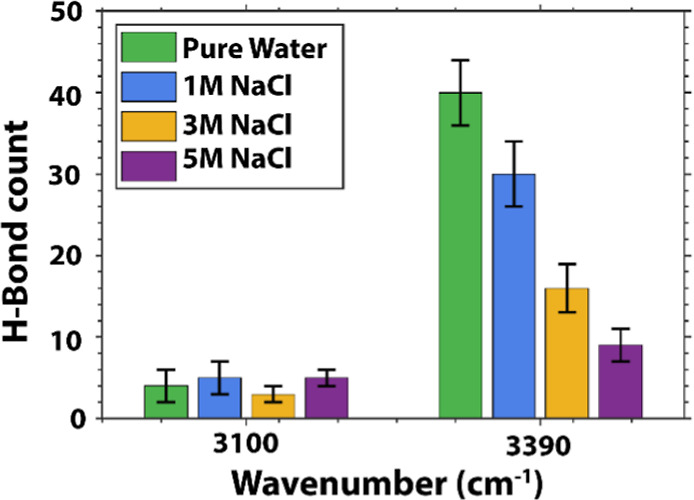
Total count of hydrogen bonds for interfacial water molecules within the entire system, corresponding to the SFG features near 3100 cm^–1^ and 3390 cm^–1^ for different NaCl concentrations.

To further interpret the changes in the interfacial populations, we investigated the spatial distribution of water molecules and ions near the interface. As shown in Figure 11a,b, the density of interfacial water within 5 Å from the surface decreases with increasing salt concentration, while the local concentration of Na^+^ increases. At 1 M concentration, the decrease in water population within the Stern layer is still marginal. This could explain the relatively insignificant changes from the pure water Imχ_Stern_^(2)^ spectrum (Figure 4b) in the Imχ_Stern_^(2)^ spectra of the low concentration regime (Figure 8a), except the weakly hydrogen-bonded ∼3480 cm^–1^ species. At 1 M, Cl^–^ counterions are still far from the silica surface (Figure 11b). As the NaCl concentration approaches its saturation concentration (6 M), the Cl^–^ counterions start to populate in the Stern layer region. Figure 11c plots the spatial distributions of all three species (water, Na^+^, and Cl^–^) at the 5 M concentration; it clearly shows that Na^+^ accumulates near the surface and water is displaced toward the bulk region and copopulated with Cl^–^ in the region adjacent to the surface-bound Na^+^ region. It is noted that even at extremely high NaCl concentrations, water molecules in the Stern layer are not completely displaced by Na^+^ ions. The water molecules within 1.5 ∼3 Å must be responsible for the ∼3220 cm^–1^ and ∼3390 cm^–1^ peaks with the positive sign in the Imχ_Stern_^(2)^ spectra and those within 2 ∼4 Å are thought to correspond to the ∼3480 cm^–1^ species with the negative sign in the Imχ_Stern_^(2)^ spectra in the high concentration regime (Figure 8c).

**Figure 11:**
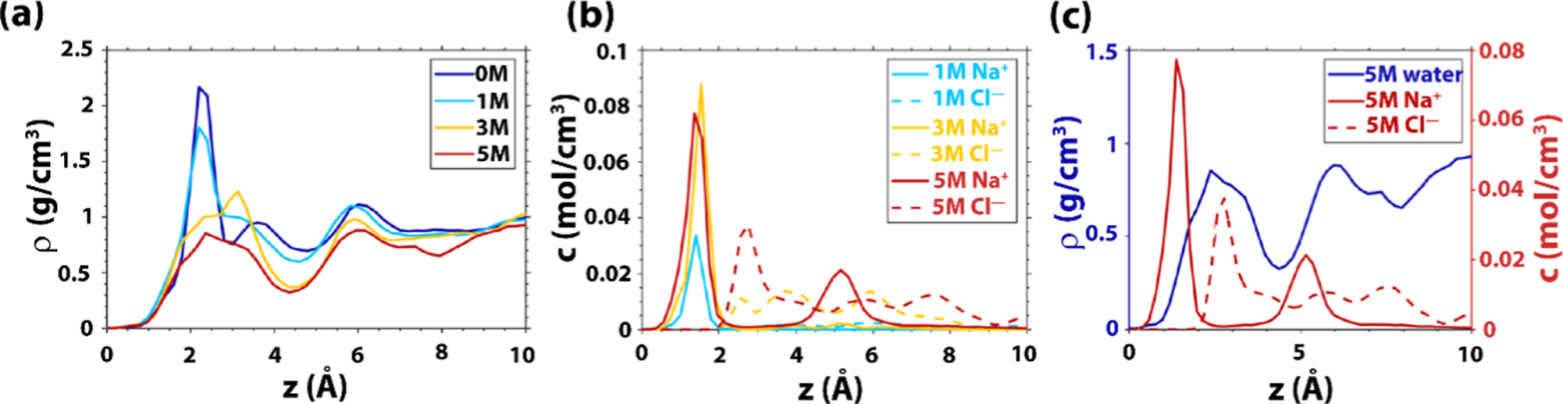
(a) Density profiles of water for varying NaCl concentrations, (b) ion concentration profiles for the same systems, and (c) spatial distribution of Na^+^ ions, Cl^–^ ions, and water molecules in the 5 M NaCl solution.

### Correlation between Hydrodynamic Slip and Interfacial Water Structure

Classical approaches that correlate slip phenomena with surface chemistry typically attribute slip to surface hydrophobicity or nanobubbles, but this pertains to nonpolar, low-energy surfaces only.^37^ Such frameworks are not suitable for hydrophilic surfaces such as silica in aqueous solutions. In the silica/water system, the slip behavior must be mostly governed by the Stern and diffuse layers, where water molecules are confined and preferentially aligned by the surface-induced electrostatic field. Deviations of water structure in these interfacial layers from bulk water will have a large impact on viscous flow near the surface. In our study, the interfacial water structure in the Stern layer revealed by the Imχ_Stern_^(2)^ spectra as well as MD simulations can be used to interpret the change in slip length as a function of ionic strength measured with AFM.

In the low ionic strength regime (≤10^–5^ M), the interfacial structure of water molecules strongly interacting with surface functional groups (silanol and silanolate) in the Stern layer does not change noticeably, except the ∼3520 cm^–1^ species corresponding to water molecules weakly hydrogen-bonded to bridging oxygen atoms exposed at the amorphous silica surface (Figures 4c and 8b). This may explain the insignificant change in the slip length in the low concentration regime (Figure 2e). It is important to note that in this study, the slip plane is at the boundary of the Stern and diffuse layers, not the water/silica interface.^62^ One might argue that a slight increase in the slip length from the pure water upon addition of 10^–5^ M NaCl could be due to the loss of the ∼3520 cm^–1^ species; but we note that such a small difference in the slip length could be due to uncertainty in the data fitting.

In the high ionic strength regime (>10^–5^ M), the interfacial water structure in the Stern layer changes significantly (Figure 8d). The Stern layer thickness increases due to the ingress and accumulation of Na^+^ ions immediately adjacent to the silica surface (Figure 11).^63^ Since Na^+^ ions attached to the surface are not mobile, the water molecules populated in the 2–4 Å region from the surface would not be highly mobile. This could be the main cause for the decrease in slip length (Figure 2e) with the increase in ionic strength in this regime.

Based on these findings, we propose conceptual models for hydrodynamic slip of water at the highly hydrophilic and charged surface. This model hypothesizes that the effective slip plane is located at the Stern/diffuse layer boundary, rather than at the solid–Stern interface. The Stern layer is composed of tightly bound water molecules and counterions (in our system, Na^+^) that interact directly with surface functional groups (e.g., silanol or silanolate), making it effectively immobile. This would result in an increase in friction factor, preventing water molecules from slipping.

Another important parameter could be the overall change in the density profile. In the absence of Na^+^ ions in the Stern layer, the density depletion region can be seen in the regions ∼3 Å and 4.5 Å from the surface (0 M case in Figure 11a). These density depleted regions are responsible for interfacial slip.^37^ Upon ingress and accumulation of Na^+^ ions in the Stern layer, the density depletion in the ∼3 Å region disappears, in fact, the overall density of this region increases since the charge-compensating ions (in our system, Cl^–^) move into the region (Figure 11c). The density depletion region is pushed further away from the surface; for example, ∼4.5 Å in Figure 11c. However, slip at this plane could still require large energy dissipation since the Cl^–^ ions in the ∼3 Å region are not highly mobile due to interactions with the Na^+^ ions in the ∼1.5 Å regime.

## Conclusions

In this study, we investigated and identified a correlation between interfacial water structure and hydrodynamic slip at the silica–water interface. SFG results showed that the MEM-derived imaginary part of second-order susceptibility of the Stern layer significantly changes with increasing NaCl concentrations, including the disappearance of the 3100 cm^–1^ and the emergence of a negative peak near ∼3480 cm^–1^. Supported by ReaxFF-based MD simulations, these spectral changes are attributed to ion-induced displacement and reorientation of water molecules within the Stern layer. In parallel, AFM measurements showed that the slip length remains nearly constant at low ionic strength but decreases sharply at higher concentrations. These results indicate that hydrodynamic slip at charged interfaces depends on the nanoscale structure of interfacial water molecules near the surface.

## Supplementary Material



## Notes

MEM-derived imaginary and real component of χ^(2)^; Image of contact angle of a hydrophilic surface; Sensitivity tests using viscosity ratios (η_eff_/η_bulk_); Experimental AFM data as a function of KCl concentration; Calculation of *g*_3_ as a function of NaCl concentration; Determination of χ^(3)^ from MEM-derived χ^(2)^ and *g*_3_; MEM-derived real and imaginary χ^(2)^ spectra of pure water in the diffuse and Stern layers; Side-view geometry of interfacial water molecules at the α-quartz (001)/water interface; Illustration of bridging oxygen at the α-quartz (001) surface; Surface charge obtained from MD simulation; Surface potential using classical electrostatic calculation (PDF)
