# Supplementary material for: Searching for a Correlation between Interfacial Liquid Structure and Hydrodynamic Slip: A Case Study of Water/Silica Interface
Source: J Phys Chem C Nanomater Interfaces. 2026 Aug 3;130(32):11347–60. doi: 10.1021/acs.jpcc.6c00760 (PMC13479788; doi:10.1021/acs.jpcc.6c00760)
Supplement: Supplementary file 1 [file jp6c00760_si_001.pdf]

Searching for a Correlation between Interfacial Liquid Structure and Hydrodynamic Slip: A Case Study of Water/Silica Interface

Juseok Choi[1], Abdul Aziz Shuvo[2], Adri C.T. van Duin[2], Bladimir Ramos-Alvarado[2], and Seong H. Kim[1*]

1) Department of Chemical Engineering and Materials Research Institute, The Pennsylvania State University, University Park, PA, 16802, USA.
2) Department of Mechanical Engineering, The Pennsylvania State University, University Park, PA, 16802, USA.

* Corresponding author: shk10@psu.edu

Supporting Information 1. MEM-derived imaginary and real component of $\chi^{(2)}$

Supporting Information 2. Image of contact angle of a hydrophilic surface

Supporting Information 3. Sensitivity tests using viscosity ratios η_{eff}/η_{bulk})

Supporting Information 4. Experimental AFM data as a function of KCl concentration

Supporting Information 5. Calculation of g_3 as a function of NaCl concentration

Supporting Information 6. Determination of $\chi^{(3)}$from MEM-derived $\chi^{(2)}$and g_3

Supporting Information 7. MEM-derived real and imaginary $\chi^{(2)}$ spectra of pure water in the diffuse and Stern layers

Supporting Information 8. Side-view geometry of interfacial water molecules at the α-quartz (001)/water interface

Supporting Information 9. Illustration of bridging oxygen at the α-quartz (001) surface.

Supporting Information 10: Surface charge obtained from MD simulation

Supporting Information 11. Surface potential using classical electrostatic calculation

Supporting Information 1. MEM-derived imaginary and real component of $\chi^{(2)}$

The complex second-order nonlinear susceptibility, $\chi^{(2)}$, was reconstructed from experimentally measured SFG intensity spectra using the maximum entropy method (MEM).[1,2] In typical SFG experiments, the detected signal intensity is proportional to the squared magnitude of the effective nonlinear susceptibility ($\chi_{eff}^{(2)}$):

$$I_{SF} \propto \left|\chi_{eff}^{(2)}\right|^2 \quad \text{(eq. S1)}$$

The experimental SFG spectrum (ω) was first preprocessed by subtracting its minimum value to ensure non-negative intensity. Then, the resulting spectrum was transformed to a normalized frequency coordinate (υ), defined as

$$\upsilon = \frac{\omega - \omega_{min}}{\omega_{max} - \omega_{min}} \quad \text{(eq. S2)}$$

A linearly interpolated continuous function $S(\upsilon)$ was constructed from the normalized data and used as the magnitude input for the MEM process.

The complex $\chi^{(2)}$ spectrum was then reconstructed as

$$\chi^{(2)}(\nu) = \frac{b e^{i\phi_\upsilon}}{1 + \sum_{m=1}^{M} a_m e^{i2\pi m\nu}} \quad \text{(eq. S3)}$$

where b^2 is a scaling factor determined self-consistently from the MEM solution, a_m is the autoregressive coefficients, m is the expansion order, and ϕ_υ is the error phase.

The error phase (ϕ_υ) describes residual phase ambiguity from MEM, ϕ_υ was defined as a linear function of frequency using reference SFG spectrum reported for the silica/water interface at pH 7[1] with points set to 68° at 3215 cm^{-1} and 105° at 3450 cm^{-1}. And the expansion order (m) was set to $M = 100$, which was sufficient to reproduce the spectral features without introducing numerical artifacts. Correlation coefficients (b^2 and a_m) were calculated by solving a Toeplitz matrix with the corresponding linear system and the constraint $a_0 = 1$.

$$R(m) = \int_0^1 S(\upsilon) e^{-i2\pi m\upsilon} d\upsilon \quad \text{(eq. S4)}$$

Finally, the reconstructed $\chi^{(2)}(\upsilon)$ was transformed back to the original wavenumber axis to obtain the real and imaginary components of $\chi^{(2)}$, as well as the corresponding $|\chi^{(2)}|^2$spectrum. Figure S1 shows a comparison between the reference SFG spectrum from Myalitsin et al.[1] and the MEM-reconstructed real and imaginary $\chi^{(2)}$ spectra.

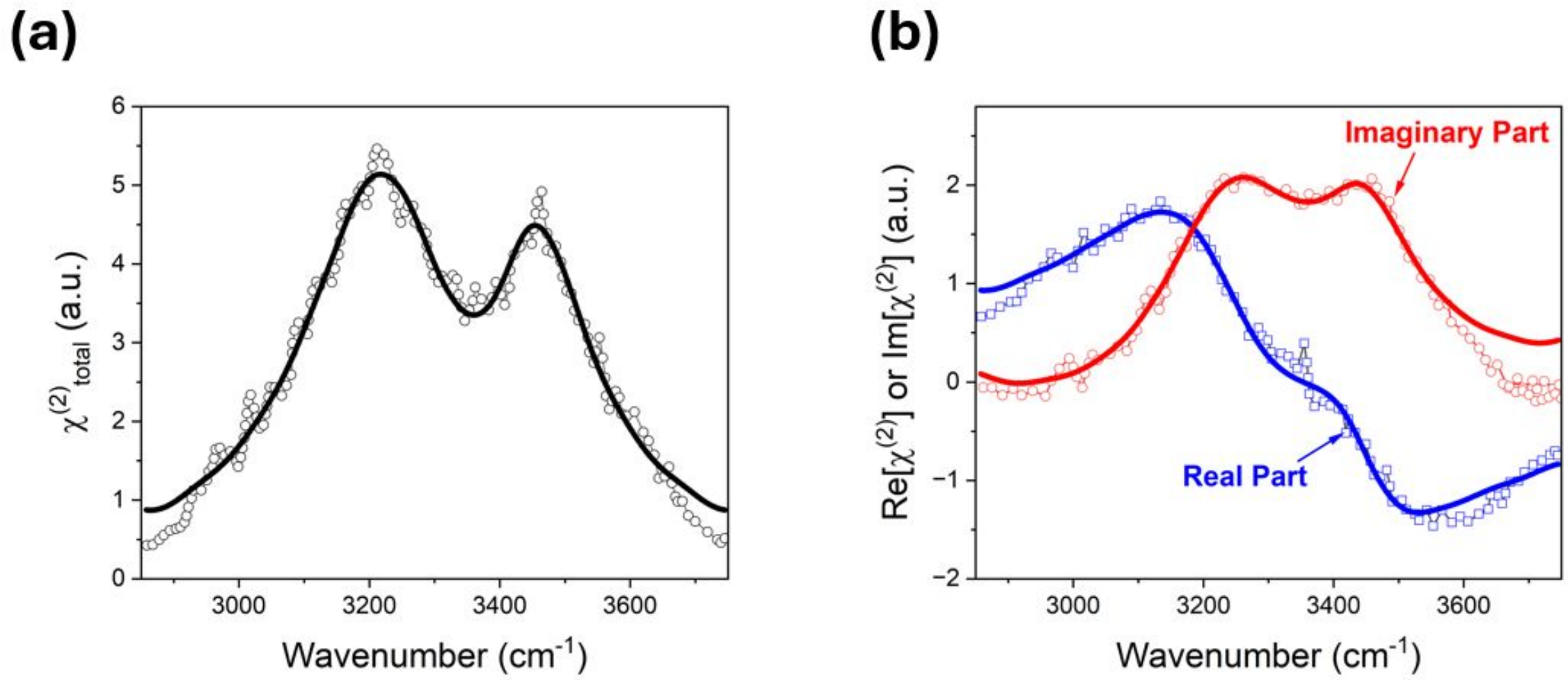

Figure S1. (a) $\chi^{(2)}_{total}$ and (b) its real and imaginary spectrum predicted by the MEM method (line) using the experimental reference data (shape) for the silica/water interface at pH 7 from Myalitsin et al.[1] The error phase (ϕ_υ) was set by a linear fit, with 68° at 3215 cm^{-1} and 105° at 3450 cm^{-1}.

Supporting Information 2. Image of contact angle of a hydrophilic surface

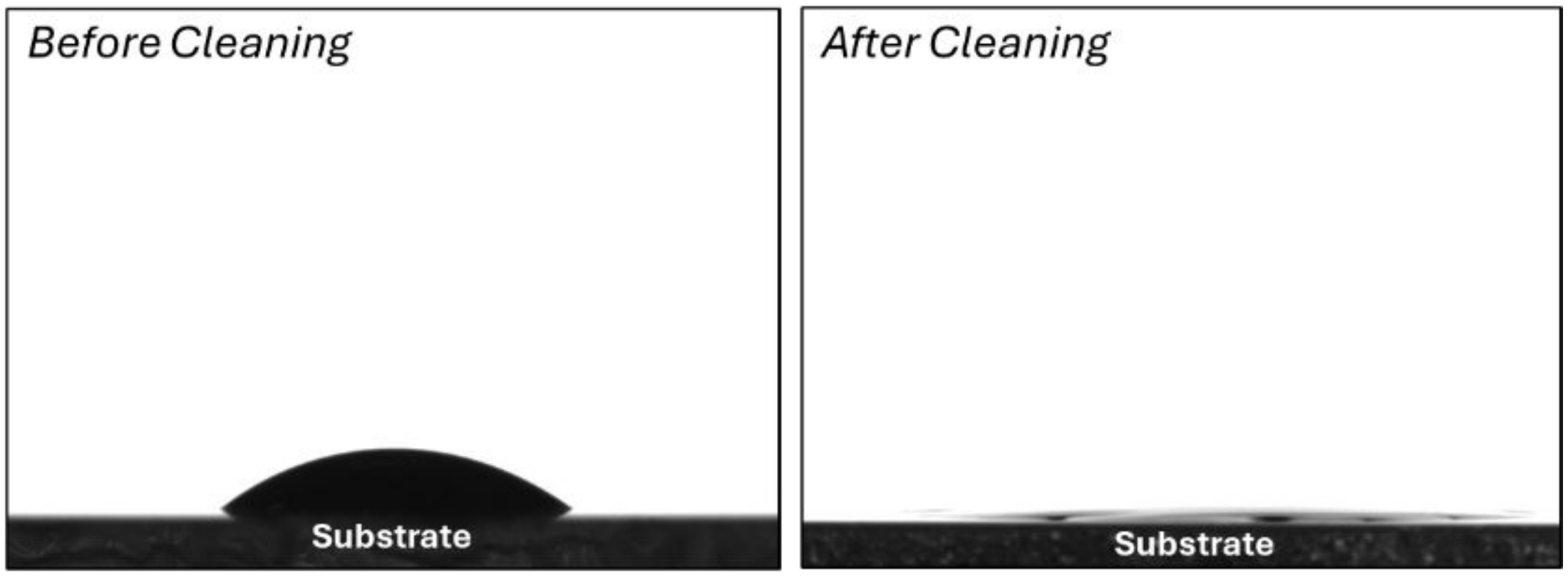

Figure S2. Contact angle images of water on a silica substrate measured before (left) and after (right) the cleaning process using a ramé-hart 260 Automated Goniometer.

Supporting Information 3. Sensitivity tests using viscosity ratios η_{eff}/η_{bulk})

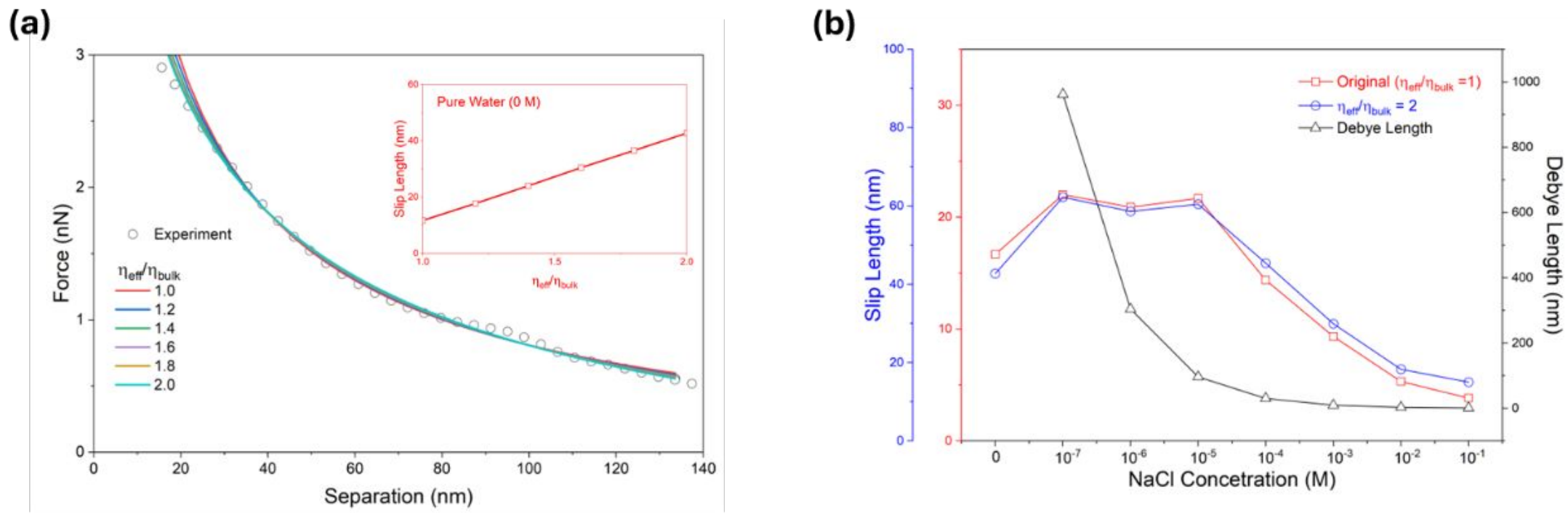

Figure S3. Evaluation of the effective interfacial viscosity (η_{eff}) and its impact on the calculated slip length. (a) Hydrodynamic force as a function of separation distance. The experimental data (open circles) are fitted with varying effective viscosity ratios (η_{eff}/η_{bulk}) ranging from 1.0 to 2.0 (solid lines). The inset demonstrates the direct linear relationship between the calculated absolute slip length and the assumed viscosity ratio in pure water (0 M). (b) Comparison of the slip length and the Debye length across different NaCl concentrations. The relative trend of the slip length, calculated with η_{eff}/η_{bulk} = 1 (red squares) and η_{eff}/η_{bulk} = 2 (blue circles)

Supporting Information 4. Experimental AFM data as a function of KCl concentration

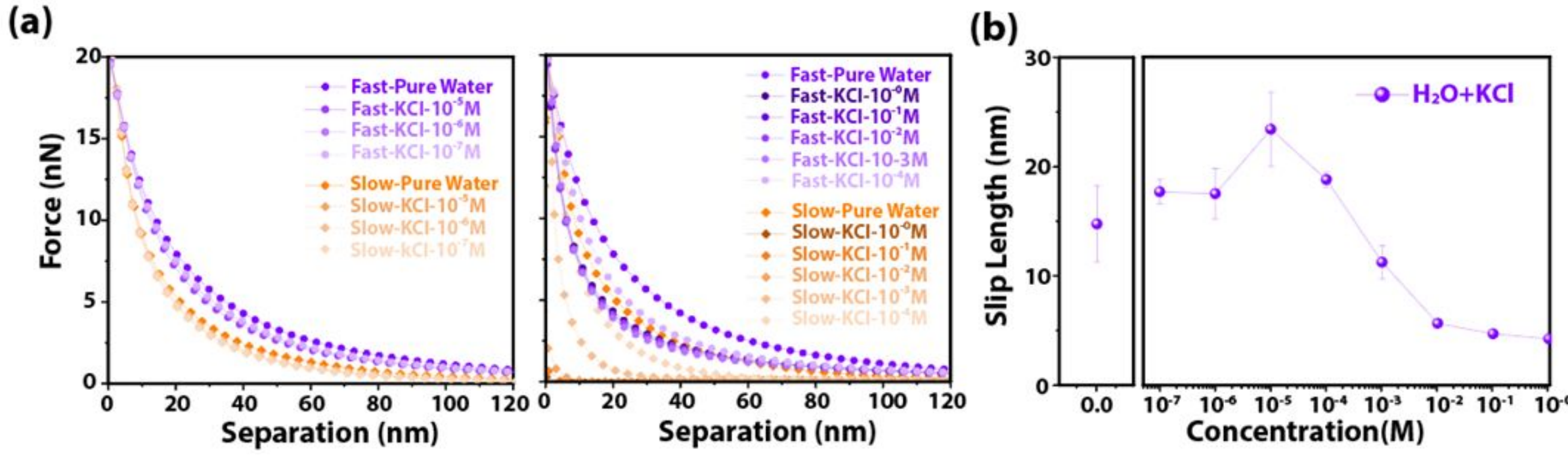

Figure S4. (a) Experimentally measured force–separation profiles during slow and fast AFM approach at varying KCl concentrations (left-side panel: low-concentration, and right-side panel: high-concentration). (b) Extracted slip length as a function of KCl concentration.

Supporting Information 5. Calculation of g_3 as a function of NaCl concentration

g_3 describes the effective interference between the surface second-order response and the bulk third-order response modulated by the interfacial electrostatic potential and phase mismatch.

$$g_3 = \Phi_0 - \frac{4ik_bT\Delta k}{e}\sum_{n=1}^{\infty}\frac{1}{(2n-1)(i\Delta k-\kappa(2n-1))}\left(\frac{e\Phi_0}{4k_bT}\right)^{2n-1} \quad \text{(eq. S5)}$$

where Φ_0, k_b, T, e, κ, and Δk are the surface potential, Boltzmann constant, temperature, elementary charge, inverse of Debye length, and phase mismatch, respectively.

The surface potential (Φ_0) was evaluated as a function of NaCl concentration using an empirical relation derived from zeta-potential measurements and the Grahame equation for the silica/water interface at pH 7, as reported in previous studies.[3]

$$\Phi_0(V) = \left(-52.42 - \frac{403.03}{1+e^{1.226*\log[NaCl]+3.026}}\right) \quad \text{(eq. S6)}$$

The Debye screening length (κ) was calculated from the electrolyte concentration assuming a symmetric 1:1 electrolyte and room temperature conditions:

$$\delta = \left(\frac{\varepsilon_0*\varepsilon_r*k_b*T}{e^2*N_A*2*[NaCl]}\right) \quad \text{(eq. S7)}$$

where ε_0, ε_r, k_b, T, e, and N_A are the vacuum permittivity, relative vacuum permittivity, Boltzmann constant, temperature, elementary charge, and Avogadro Number, respectively.

The phase mismatch (Δk) was calculated using the experimental laser geometry, refractive indices (n) of silica and water at the visible, infrared, and sum-frequency wavelengths (λ), and Snell's law for transmission geometry. But, the sign of each momentum need to be based on the vector direction with respect to the surface normal.

$$\Delta k = |k_{SF} - (k_{Vis} + k_{IR})| \quad \text{(eq. S8)}$$

$$k_i = \frac{2\pi n \cos\theta_{refraced}}{\lambda} \quad \text{(eq. S9)}$$

Using these concentration-dependent electrostatic parameters and fixed optical parameters for the experimental configuration, $g_3$was calculated as a function of NaCl concentration in a corresponding wavenumber. Figure S4 shows the calculated g_3 values at 3200 cm^{-1} and 3400 cm^{-}

[1], demonstrating nearly identical concentration-dependent trends at the two wavenumbers. This result indicates that g_3 is governed by concentration-dependent electrostatic parameters, such as surface potential and Debye length, rather than by wavenumber-dependent optical parameters, such as phase mismatch.

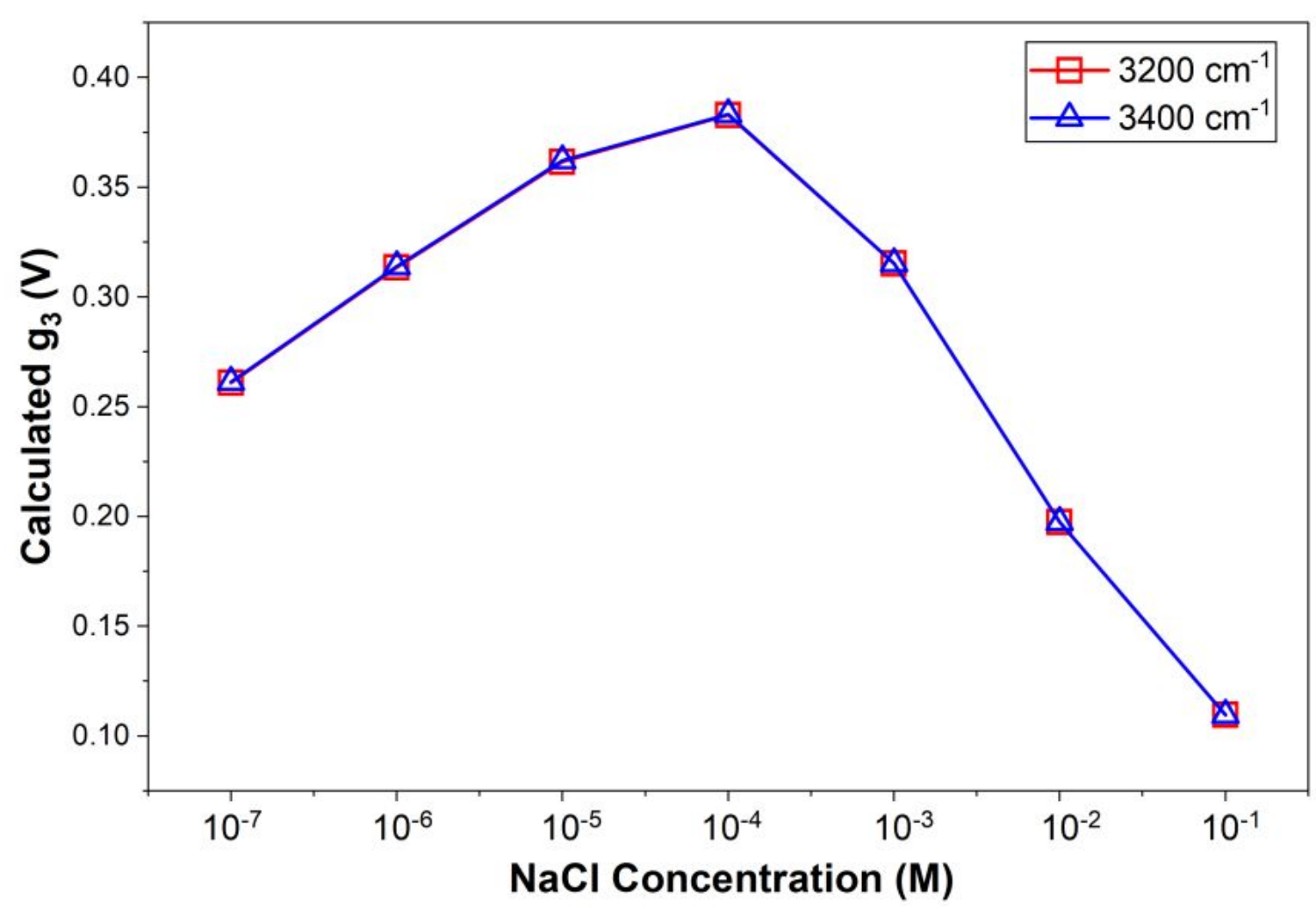

Figure S5. Calculated g_3 as a function of NaCl concentration at 3200 cm^{-1} and 3400 cm^{-1}. The concentration-dependent trends of g_3 at the two wavenumbers are nearly identical, indicating that g_3 is governed by concentration-dependent parameters (e.g., surface potential or Debye length) rather than wavenumber-dependent parameters (e.g., phase mismatch).

Supporting Information 6. Determination of $\chi^{(3)}$ from MEM-derived $\chi^{(2)}$ and g_3

The third-order nonlinear susceptibility, $\chi^{(3)}$, was determined by combining the MEM-reconstructed complex second-order susceptibility with the calculated g_3 parameter.

To extract $\chi^{(3)}$, MEM-derived real and imaginary $\chi^{(2)}_{total}$ spectra were obtained at two different NaCl concentrations, C_1 and C_2. By assuming $\chi^{(2)}_{Stern}$ is identical at the two concentrations, subtracting the two spectra isolates $\chi^{(3)}$ contribution. Because both $\chi^{(3)}$ and g_3 are complex numbers, so simple scalar addition or subtraction is not applicable. Instead, the complex $\chi^{(3)}$ was obtained by solving Eq. S9.

$$\chi^{(2)}_{total} = \chi^{(2)}_{Stern} + \chi^{(2)}_{Diffuse} = \chi^{(2)}_{Stern} + \chi^{(3)} * g_3 \qquad \text{(eq. S10)}$$

$$\begin{cases} \mathbb{Re}\left(\chi^{(2)}_{total,C_1}\right) - \mathbb{Re}\left(\chi^{(2)}_{total,C_2}\right) = \mathbb{Re}\left(\left(g_{3,C_1} - g_{3,C_2}\right)\right) \cdot \mathbb{Re}\left(\chi^{(3)}\right) - \mathbb{Im}\left(\left(g_{3,C_1} - g_{3,C_2}\right)\right) \cdot \mathbb{Im}\left(\chi^{(3)}\right) \\ \mathbb{Im}\left(\chi^{(2)}_{total,C_1}\right) - \mathbb{Im}\left(\chi^{(2)}_{total,C_2}\right) = \mathbb{Re}\left(\left(g_{3,C_1} - g_{3,C_2}\right)\right) \cdot \mathbb{Im}\left(\chi^{(3)}\right) + \mathbb{Im}\left(\left(g_{3,C_1} - g_{3,C_2}\right)\right) \cdot \mathbb{Re}\left(\chi^{(3)}\right) \end{cases}$$

(eq. S11)

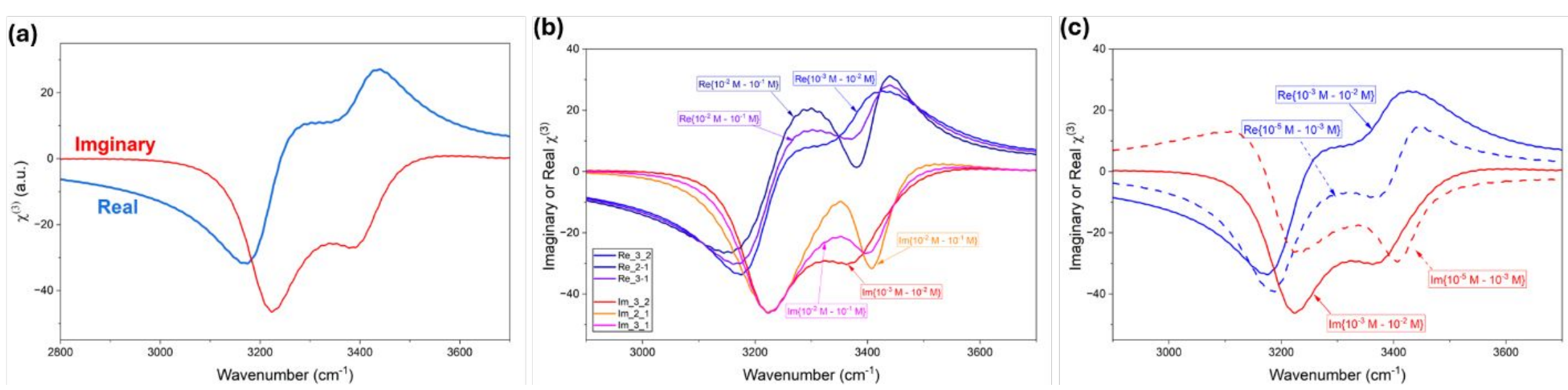

Figure S6. (a) Real and imaginary components of $\chi^{(3)}$ calculated by subtracting the total complex spectra of water at 10^{-3} and 10^{-1} M NaCl and dividing by the corresponding g_3 function, Consistency validation of extracted $\chi^{(3)}$ with 10^{-3}, 10^{-2}, and 10^{-1} M data. Real and imaginary parts of $\chi^{(3)}$ spectra extracted using adjacent concentration pairs (b) in the same regime (10^{-3} – 10^{-2} M, 10^{-3} – 10^{-1} M, and 10^{-2} – 10^{-1} M) and (c) in the different regime (10^{-5} – 10^{-3} M)

Supporting Information 7. MEM-derived real and imaginary $\chi^{(2)}$ spectra of pure water in the diffuse and Stern layers

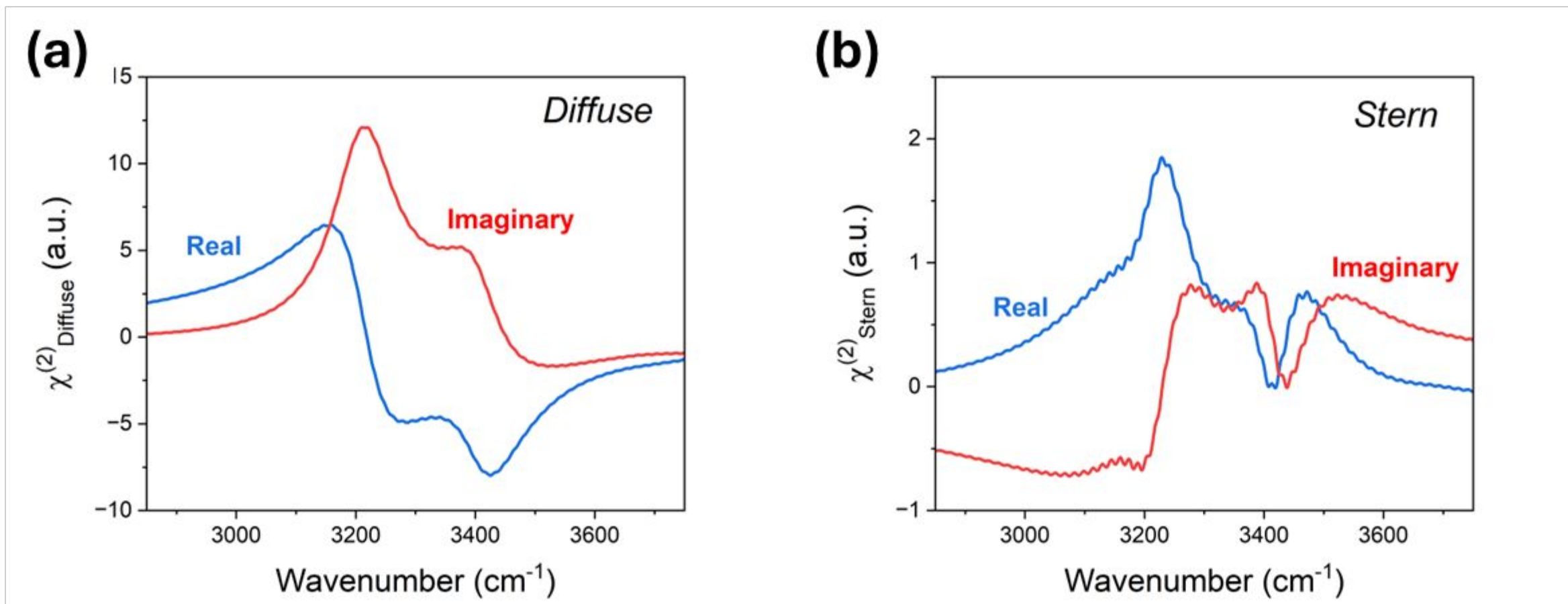

Figure S7. MEM-derived real and imaginary components of $\chi^{(2)}$ of (a) Diffuse and (b) Stern layer of a silica/pure water (0 M NaCl) interface

Supporting Information 8. Side-view geometry of interfacial water molecules at the α-quartz (001)/water interface

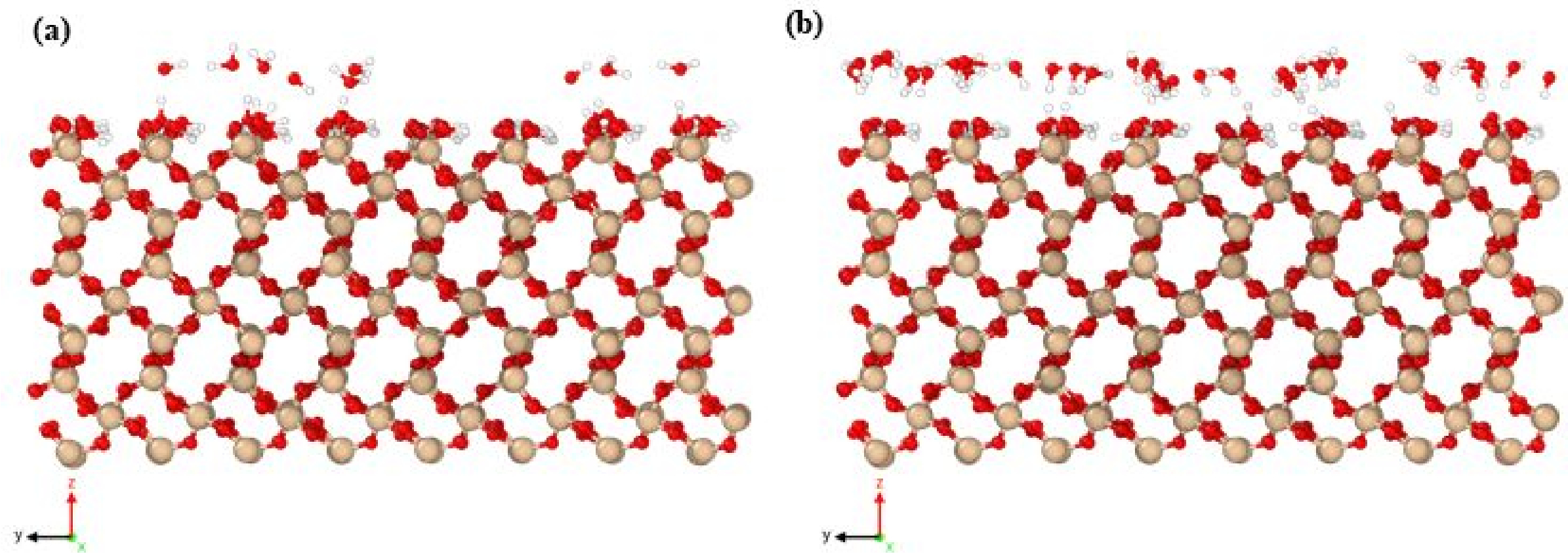

Figure S8. Side views (x-axis projection) of interfacial water molecules at the α-quartz (001)/water interface corresponding to the molecular configurations shown in Figure 5.

Supporting Information 9. Illustration of bridging oxygen at the α-quartz (001) surface.

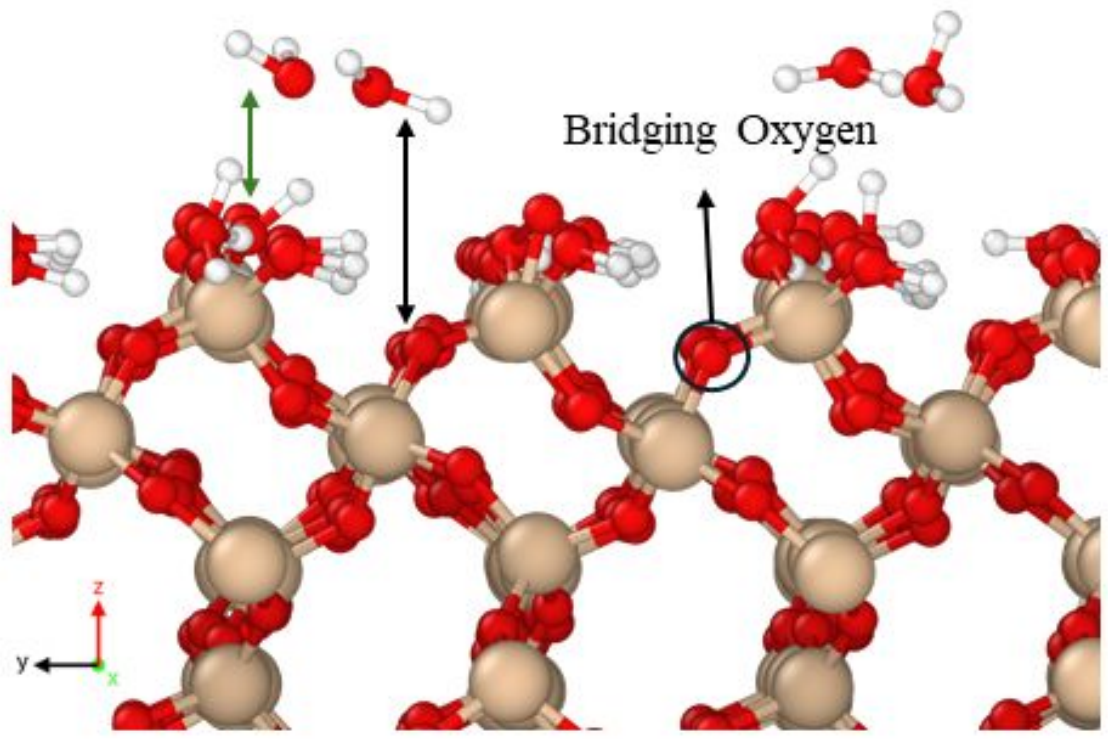

Figure S9. Illustration of bridging oxygen at the α-quartz (001) surface.

Supporting Information 10. Surface charge obtained from MD simulation

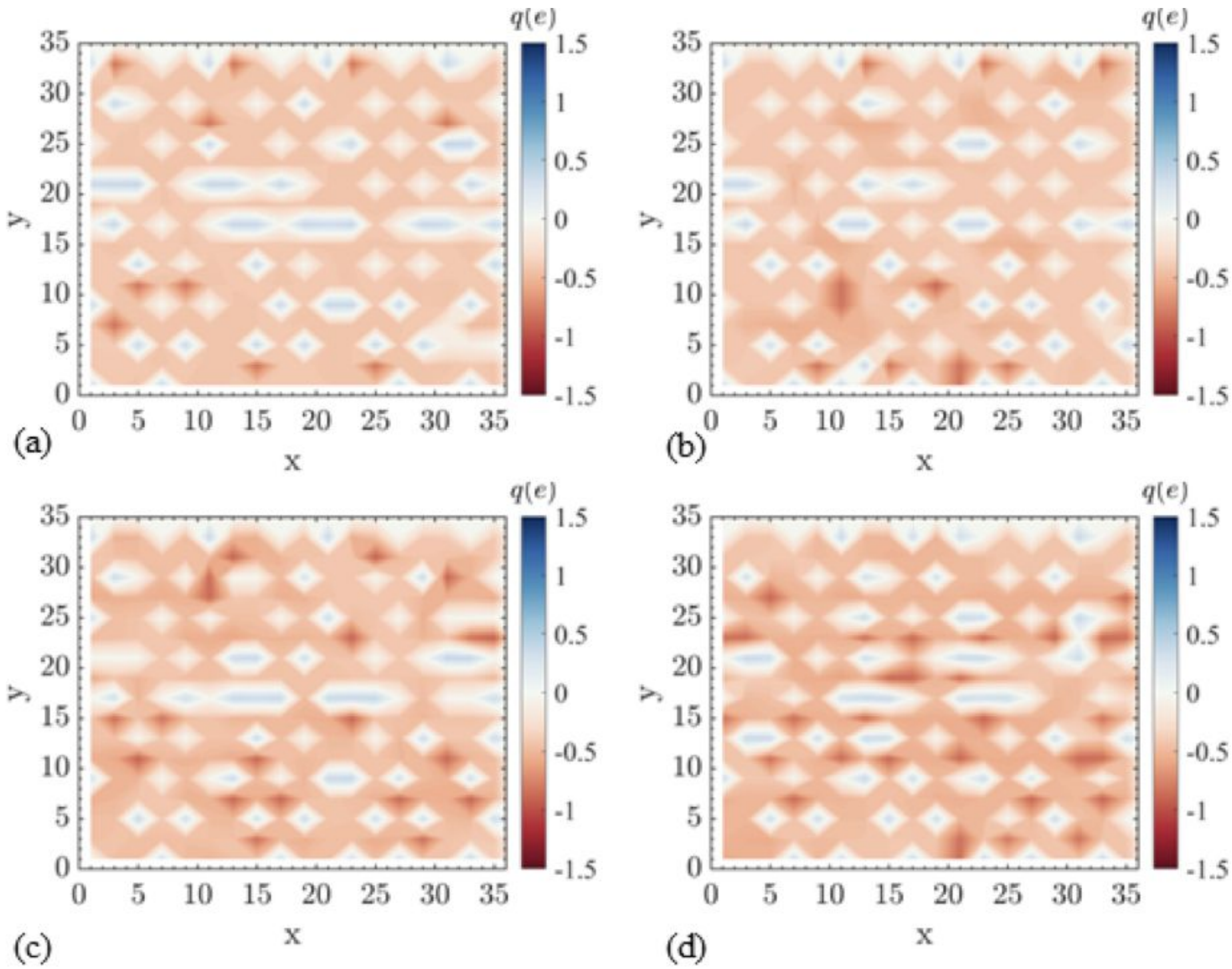

Figure S10: Surface charge contour at 0.5 Å from the bottom of the solid slab (a) pure water, (b) 1M, (c) 3M, and (d) 5M concentration of NaCl.

Supporting Information 11. Surface potential using classical electrostatic calculation

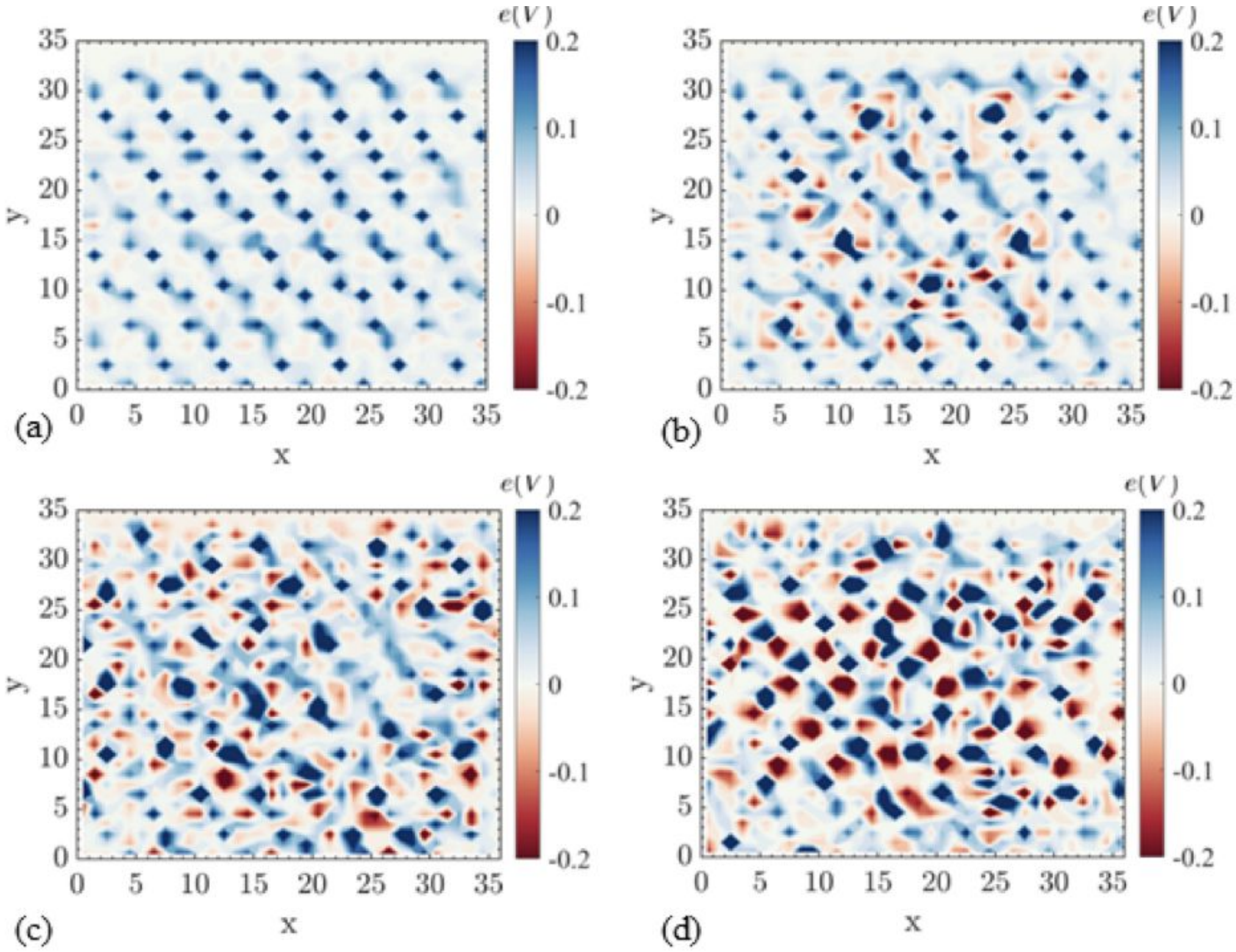

Figure S11. MD-calculated surface potential maps calculated 5 Å above the interface for various electrolyte concentrations: (a) pure water, (b) 1 M, (c) 3 M, and (d) 5 M NaCl solution.

References

1. Myalitsin, A.; Urashima, S.-h.; Nihonyanagi, S.; Yamaguchi, S.; Tahara, T. Water Structure at the Buried Silica/Aqueous Interface Studied by Heterodyne-Detected Vibrational Sum-Frequency Generation. *The Journal of Physical Chemistry C* **2016**, *120* (17), 9357-9363. DOI: 10.1021/acs.jpcc.6b03275.

2. Rehl, B.; Gibbs, J. M. Role of Ions on the Surface-Bound Water Structure at the Silica/Water Interface: Identifying the Spectral Signature of Stability. The Journal of Physical Chemistry Letters 2021, 12 (11), 2854-2864. DOI: 10.1021/acs.jpclett.0c03565.

3. Tan, J.; Wang, M.; Zhang, J.; Ye, S. Determination of the Thickness of Interfacial Water by Time-Resolved Sum-Frequency Generation Vibrational Spectroscopy. Langmuir 2023, 39 (50), 18573-18580. DOI: 10.1021/acs.langmuir.3c02906.
